# Older forensic mental healthcare patients in England: demographics, physical health, mental wellbeing, cognitive ability and quality of life

**DOI:** 10.3310/nihropenres.13248.1

**Published:** 2022-02-03

**Authors:** Jack Tomlin, Kate Walker, Jen Yates, Tom Dening, Birgit Völlm, Chris Griffiths

**Affiliations:** 1 School of Law and Criminology, University of Greenwich, London, UK; 2 Innovation and Research Department, Northamptonshire Healthcare NHS Foundation Trust, Northampton, UK; 3 Division of Psychiatry and Applied Psychology, School of Medicine, University of Nottingham, Nottingham, UK; 4 Department of Forensic Psychiatry, University Hospital Rostock, Rostock, Germany

**Keywords:** Forensic mental health; older patients; quality of health; mental wellbeing; recovery

## Abstract

**Background:**

Older individuals (e.g., 55 years and over) constitute a growing proportion of the forensic mental health patient population. As a group, they are vulnerable to health outcomes similar to other individuals with serious mental disorders of the same age; however, these concerns can be compounded by complex forensic-related care backgrounds and clinical presentations, lengthy periods of time spent in prison or psychiatric hospitals, substance use histories, and crime perpetration or victimisation. The healthcare needs and strengths of this group are not well understood.

The aim of this study was to identify and describe the demographic, physical health, mental wellbeing, cognitive ability, and quality of life profiles of older forensic patients in community, low, medium, and high security settings in England.

**Methods:**

A cross-sectional mixed-methods study design was used. N=37 forensic patients aged 55 years and over were interviewed and completed questionnaires. Data were also collected from patient records.

**Results:**

Most patients were male and were diagnosed with psychosis. The most frequently committed index offence types were violent offences. Patients were prescribed 7.6 medications on average and had average anticholinergic effect on cognition scores of 2.4. Nearly half the sample had diabetes, with an average BMI score of 31.7 (indicating obesity). Possible cognitive impairment was identified in 65% of the sample. Patients’ assessments of their recovery-related quality of life and mental wellbeing did not differ from published UK general population values. Assessments of quality of life were positively correlated with the ability to undertake everyday activities and cognitive performance.

**Conclusions:**

We suggest that forensic services are well-placed to provide holistic mental and physical care to this group but that they should co-develop with patients a greater range of age-appropriate meaningful activities that are mindful of mobility issues and consider implementing more cognition-based and physical health interventions.

## Plain language summary

Forensic mental health services provide care for people who have committed a crime or are at risk of harm to themselves or others. This care takes many forms, including mental health support, physical health care, and assistance to re-enter the community after spending time in secure mental health hospitals. Due to changes in the general population, there is a growing number of patients over the age of 55 in care. Despite this, we do not know enough about the healthcare needs of this older patient group and how these needs differ from younger patients. This study aimed to find out more about this patient group.

In the current study, thirty-seven forensic patients aged 55 years and older were recruited from low, medium and high security hospitals and community care. These patients completed six questionnaires and participated in an interview with a researcher. Patients were asked about their physical health, mental wellbeing, cognitive ability, and quality of life. Information about medical diagnoses and socio-demographic backgrounds were collected from hospital records.

The study found that these patients had complex health needs. Most patients were diagnosed with psychosis (e.g., schizophrenia), were men, and had committed a violent criminal offence. On average patients were prescribed 7.6 medications, many of which carry significant side effects. Nearly half of the patients had diabetes, and most patients were obese or overweight. Quality of life scores were lower for patients who also reported having problems undertaking everyday activities and patients with cognitive impairment. Patients subjectively rated their mental wellbeing and quality of life the same as the general population’s ratings as reported in other studies. We suggest that services should work with patients to develop a greater number of age-appropriate activities and interventions aimed at improving cognitive and physical health.

## Introduction

Providing support, care and treatment for forensic patients that is responsive to their individual needs and strengths is a core tenet of the recovery approach ( Simpson & Penney, 2018). Recognising this requires investigating the individual profiles of patients in forensic mental health services and how patients’ backgrounds, characteristics, experiences and perspectives differ. This approach can be seen in recent efforts to develop services and interventions responsive to the lived experiences, strengths and needs of, amongst others, women patients ( de Vogel & Nicholls, 2016), culturally and ethnically diverse patients ( Hui, 2017), Deaf patients ( Wakeland *et al.*, 2019), and older patients ( Solares *et al.*, 2020). This latter group was the focus of the ENHANCE study ^1^, which investigated the demographics, health-related quality of life, recovery rated quality of life, mental wellbeing, cognitive ability, wellbeing and secure hospital restrictiveness rating profiles of forensic mental healthcare patients aged 55 and over.

Forensic mental healthcare patients aged 50 and over constitute about 20% of the UK forensic mental healthcare inpatient population ( Di Lorito *et al.*, 2019; Di Lorito *et al.*, 2018b). This proportion is likely to increase as the population ages ( World Health Organization, 2017); indeed, the number of forensic inpatients over 65 in Scotland increased by 50% and those aged 56–65 by 27% between 2013 and 2019 ( Scottish Government, 2021). The physical and mental healthcare needs of this group are complex as often comorbidities are prevalent ( Natarajan & Mulvana, 2017). Many forensic mental healthcare patients have biographies characterised by placements in psychiatric institutions and prisons, and have experiences of substance abuse and long-term serious mental and physical illnesses for which they may have not received appropriate treatment or management ( Centre for Mental Health, 2010; Centre for Mental Health, 2013). This constellation of factors means that many forensic patients experience an ‘accelerated aging’, presenting with a level of health need at, for example, 50 years old that would be equivalent to an average member of the general public at 60 years ( Merkt *et al.*, 2020).

Compared to younger adult mental healthcare patients, older individuals are more likely to have a higher number of unmet health needs and to experience fewer improvements in their health over time ( Das *et al.*, 2011; Das *et al.*, 2012; Girardi *et al.*, 2018). Older patients are more likely to be diagnosed with depression, organic brain syndrome, or delusional disorder ( Coid *et al.*, 2002). Disabling health issues are more prevalent in older patients; these include cognitive decline, mobility problems and sensory impairment ( Di Lorito *et al.*, 2019). The number of medications given to older patients has been found to double throughout placement in secure hospitals, highlighting a decline in health ( Lightbody *et al.*, 2010). Investigating and documenting the disparities between younger and older mental healthcare patients can help shape service provision, develop responsive and appropriate interventions, and address structural disparities in health and wellbeing outcomes ( Hui *et al.*, 2021).

## Aims and rationale of this study

Despite the growing research and clinical interest in older adult forensic mental health patients there remains a paucity of research data. The aims of this study were to better understand the profiles of this patient group and investigate factors associated with quality of life, recovery, and wellbeing. The current study describes the quality of life, physical health, mental wellbeing, cognitive ability, demographics, and experiences of restrictiveness in secure care of 37 forensic mental healthcare patients, aged 55 and older, from community and low, medium and high security inpatient settings in England.

The research aims were co-authored with the ENHANCE study’s Lived Experience Advisory Panel (LEAP) (a group made up of people with lived experience of mental health issues and forensic mental healthcare service use). These aims were to investigate whether physical health, health-related quality of life, and recovery-related quality of life were correlated with each other in this population, and whether these constructs were associated with: mild cognitive impairment, age, length of stay in secure care, amount of leave (for inpatients), experiences of secure hospital restrictiveness, and treatment setting (i.e. community or low, medium or high security in-patient hospital). The study further investigated to what extent patients’ scores on measures of mild cognitive impairment, recovery-related quality of life, mental wellbeing and experiences of restrictiveness in secure care differed from population norms or samples described in research literature.

## Methods

### Participants

Participants were patients aged 55 and above recruited from a range of forensic mental health settings in England, from high secure in-patient care to community forensic mental health services. The ENHANCE study also included interviews with staff members from these services (reported separately) and patients known to these professionals were invited to take part. All participants were able to provide informed consent to complete questionnaires and to be interviewed.

Ethical approval was granted by the NHS Health Research Authority (IRAS: 258016; REC: 19/EM/0350). Funding was provided by the National Institute for Health Research [PB-PG-1217-20028].

### Data collection

Data collection took place between March 2020 and September 2021 across eight National Health Service (NHS) trusts. All patients were recruited through members of patients’ healthcare teams. In total, semi-structured interviews were conducted with 37 patients. Interviewing took place in person ( *n*=10), via video call ( *n*=26), or over the phone ( *n*=1). All patients gave informed consent, with written consent taken from those interviewed face-to-face, and verbal recorded consent from those interviewed via video call or phone. Both methods of recording consent were approved by the relevant ethics committee.

The interviews explored experiences of care and treatment, and what patients felt were barriers and facilitators to recovery. Clinical, legal and demographic data ( Tomlin *et al.*, 2022) were extracted from patient clinical records by principal investigators based at each recruitment site. Legal data included length of stay in the service, nature of the index offence(s) and Mental Health Act 1983 status. Index offences were categorised according to the UK Home Office Offence Classification Index. Where a patient had more than one index offence, we report the most severe as indicated by the Home Office Crime Severity Score.

Clinical data included ICD-10 diagnoses, body mass index (BMI), lists of physical health conditions and medication data (total number of drugs currently prescribed, number of psychotropic drugs currently prescribed, and Anticholinergic Effects on Cognition scores ( Bishara *et al.*, 2017)). Medications were included if prescribed for regular consumption. As required ( *pro re nata* or prn) prescriptions were not counted as it would not be possible to ascertain how much of the drug had actually been administered.

In addition to the interviews, patients completed six questionnaires:

1. The Short Warwick-Edinburgh Mental Wellbeing Scale (SWEMWBS; Tennant *et al.*, 2007). This is an overall mental wellbeing measure, consisting of seven self-report questions and Likert scale responses. Higher total ‘metric scores’ indicate better mental wellbeing.
2. The EQ-5D-5L ( Devlin *et al.*, 2018) is a measure of overall health-related quality of life. It has one self-report question asking for a ‘health today’ score (a higher score indicates better health), and five self-report questions targeting the domains: ‘mobility’, ‘self-care’, ‘usual activities’, ‘pain and discomfort’, and ‘anxiety and depression’ (higher scores on these domains indicate a greater number of problems). Likert scale responses are used. Researchers calculate an ‘index score’, which summarises responses across these five domains.
3. The Recovering Quality of Life measure (ReQoL-10; Keetharuth *et al.*, 2018) is an overall recovery-related quality of life questionnaire. This has 10 self-report questions with Likert scale responses. Higher scores indicate more positive quality of life.
4. The Cambridge Contextual Reading Test – Short Version (Short CCRT; Beardsall, 1998). This is a reading task measure of premorbid IQ, wherein respondents must read sentences aloud that include difficult to pronounce words. Scores are calculated by noting the number of incorrectly pronounced words, such as ‘bouquet’, ‘thyme’ or ‘subtle’. Higher scores indicate better performance.
5. The Montreal Cognitive Assessment (MoCA; Nasreddine *et al.*, 2005) is a measure of cognitive impairment. It captures respondents’ performance across the domains: attention and concentration, executive functions, memory, language, visuoconstructional skills, conceptual thinking, calculations, and orientation. A total score out of 30 can be calculated, with higher scores indicating better performance. A score <26 indicates possible mild cognitive impairment.
6. The Forensic Restrictiveness Questionnaire (FRQ; Tomlin *et al.*, 2019). This measures inpatients’ experiences of the restrictiveness of secure inpatient care across 15, self-report Likert scale items. Higher scores indicate greater levels of perceived restrictiveness.

Only the quantitative results are reported in this article (see Walker *et al.*, 2022a; Walker *et al.*, 2022b for the results of the qualitative interviews).

### Data analysis

A required minimum sample size of *N*=36 was calculated based on an *a priori* power calculation using G*Power ( *r* = 0.5, *p* = 0.05, power = 0.95) ( Faul *et al.*, 2007).

Three percent of the questionnaire data were missing, largely due to nine patients not completing the Short CCRT, reporting difficulties with eyesight, reading level or not wanting to complete this. Eight percent of the demographic, legal and clinical data were missing due to recording issues in patient files. Pairwise deletion was used to handle missing data. IBM’s statistical package for the social sciences (SPSS) software v.27 was used. The distribution of questionnaire response data was assessed with the Shapiro-Wilk statistic and most variables were non-normally distributed. To account for this, non-parametric methods were used.

The internal consistency of the questionnaires used in the study was assessed prior to analysis. This suggested all questionnaires were appropriate to use: SWEMWBS, α= .886, *n*=36; EQ-5D-5L, α= .871, *n*=36; ReQoL, α= .859, *n*=36; FRQ, α= .945, *n*=27; and MoCA, α= .660, *n*=34. The alpha value (α) for the MoCA was lower than for the other measures and much of the literature ( Nasreddine *et al.*, 2005) but other studies ( Bernstein *et al.*, 2011) have reported values lower than α=.7 (the recommended cut-off for assuming adequate internal consistency; Bland & Altman, 1997). Spearman’s RHO ( *ρ*) was used to assess correlations ^2^. One-sample t-tests with SPSS v.27’s Bias corrected and accelerated (BCa) bootstrapping function with 1000 samples was used to compare mean questionnaire scores to population norms and samples from the literature. Statistical significance was set at *p*=<0.05; effect sizes are reported where appropriate. 

## Results

### Descriptive statistics

Table 1 presents the demographic, clinical and legal profiles of the patient group. Table 2 and Table 3 give an overview of the mental health and physical diagnoses given to patients in the sample. These show that participants were mostly men (92%), of white British ethnicity (81%), with a mean age of 60 years. Median length of stay in current institution was 1404 days (approximately 45 months). The most frequently diagnosed mental disorders were schizophrenia, schizotypal, and delusional disorders (60%), any type of personality disorder (41%), and then mental and behavioural disorders due to psychoactive substance use and mood (affective) disorders (both at 16.2%) ^3^. Scores for each questionnaire are presented in Table 4.

**Table 1.** Demographic, clinical and legal characteristics of the sample.

Characteristic	Frequency / mean / median	% / SD / 25th & 75th
**Age** ( *n*=37)	*Mn*= 59.8	*SD*= 3.9
**Sex** ( *n*=37)		
- Men	34	92
- Women	3	8
**Ethnicity** ( *n*=37)		
- White	30	81
- Black, African, Caribbean, or Black British	6	16
- Mixed or multiple ethnic group	1	3
**Mental Health Act 1983 section** ( *n*=37)		
- No legal section (community treatment)	5	14
- s. 3 (civil admission for treatment)	3	8
- s. 37/41 (hospital order and restriction order)	11	30
- s. 37/42 (hospital order and lifted restriction order)	1	3
- s. 41 (treatment in the community and restriction order)	2	5
- s. 41 (5) (notional hospital order)	2	5
- s. 42 (treatment in the community and lifted restriction order)	2	5
- s. 45 (A) (hybrid treatment order)	1	3
- s. 47/49 (prison transfer and restriction order)	9	24
- s. 117 (aftercare following discharge)	1	3
**Index offence** ( *n*=37)		
- (Attempted) Murder / Manslaughter	11	30
- Violence against the person	8	21
- Sexual offences	8	21
- Robbery	2	5
- Possession of weapons	1	3
- Threatening to destroy or damage property	1	3
- No offence	6	16
**Setting** ( *n*=37)		
- Community	10	27
- Low secure	8	22
- Medium secure	9	24
- High secure	10	27
**Length of stay in days** ( *n*=32)	*Mdn*= 1404	25 ^th^= 469; 75 ^th^= 3803
**Number of current prescribed medications** ( *n*=37)	*Mn*= 7.6	*SD*= 4.4
**Number of current prescribed psychotropic medications** ( *n*=37)	*Mn*= 2.1	*SD* = 1.5
**Anticholinergic effect of medications on cognition scores** ( *n*=37)	*Mn*= 2.4	*SD* = 2.1
**Body Mass Index** (BMI; *n*=30)	*Mn*= 31.7	*SD* = 4.5
**Possible** **mild cognitive impairment according to MoCA** ( *n*=34)		
- Yes	22	65
- No	12	35

Notes. Percentages of observed values, i-e- excluding missing values. *Mdn*, median; *Mn*, mean; *SD*, standard deviation; 25 ^th^ and 75 ^th^ percentiles.

**Table 2.** Mental health diagnoses in the sample.

Diagnoses ordered by ICD-10 categories	Frequency	%
Organic, including symptomatic, mental disorders	1	2.7
Mental and behavioural disorders due to psychoactive substance use	5	13.5
Schizophrenia, schizotypal and delusional disorders	22	59.5
Mood [affective] disorders	6	16.2
Neurotic, stress-related and somatoform disorders	3	8.1
Personality disorders (Any)	15	40.5
- Dissocial	5	13.5
- Dependent	3	8.1
- Avoidant (anxious)	5	13.5
- Emotionally Unstable	4	10.8
- Paranoid	4	10.8
- Schizoid	2	5.4
- Antisocial	4	10.8
- Borderline	3	8.1
- Mixed Personality Disorder	2	5.4
Disorders of sexual preference	1	2.7
Disorders of psychological development	2	5.4

Notes. Observations greater than 37 and percentages greater than 100 as most patients had multiple diagnoses. *N*=37.

**Table 3.** Physical health burden of the sample.

Physical diagnoses	Frequency	%
Diabetes	18	48.7
Cardiovascular and circulatory system	14	37.8
High cholesterol (e.g. hypercholesterolemia, hyperlipidaemia, raised triglycerides)	7	18.9
Chronic obstructive pulmonary disease (COPD)	6	16.2
Visual impairment	5	13.5
Asthma	4	10.8
Vitamin D deficiency	4	10.8
Diseases of the musculoskeletal system and connective tissue	3	8.1
Hearing loss	1	2.7
Impaired Physical Mobility	1	2.7

Notes. *N*=37.

**Table 4.** Questionnaire scores of the sample.

Questionnaire	Mean	SD
SWEMWBS Metric Score ( *n*=36)	23.5	6.3
ReQoL Total Score ( *n*=36)	25.7	8.9
EQ-5D-5L Index Value ( *n*=36)	0.6	0.4
FRQ Total Score ( *n*=27)	32.9	15.7
MoCa Total Score ( *n*=34)	23.5	3.8
Short CCRT ( *n*=28)	19.3	4.7

### Mental wellbeing

Mental wellbeing (SWEMWBS) was significantly positively associated with recovery-related quality of life (ReQoL) ( *ρ*= .773), and ‘health today’ ( *ρ*= .486) ^4^ as assessed by the EQ-5D-5L. It was negatively correlated with the depression and anxiety domain ( *ρ*= -.348) of the EQ-5D-5L and was trending towards a significant relationship with the ‘usual activities’ domain of the same measure (meaning fewer problems in these domains; *ρ*= -.324, p=.054). A negative correlation was also observed for inpatient perceptions of restrictiveness in care ( *ρ*= -.481). The association between mental wellbeing and mild cognitive impairment was trending towards significance in a negative direction ( *ρ*= -.320, *p*=.065).

### Recovery-related quality of life

Recovery-related quality of life positively correlated with the EQ-5D-5L ‘health today’ domain ( *ρ*= .627) and its overall index value ( *ρ*= .362). Recovery-related quality of life was negatively correlated with ‘mobility’ ( *ρ*= -.340), ‘usual activities’ ( *ρ*= -.551), ‘depression and anxiety’ domains on the EQ-5D-5L ( *ρ*= -.408) implying fewer problems on these domains, and (for inpatients) experiences of restrictiveness ( *ρ*= -.608). The association between recovery-related quality of life and mild cognitive impairment was also significant in a negative direction ( *ρ*= -.377).

### Health status and perceptions of physical wellbeing

Nearly half (49%) our sample were diagnosed with diabetes, 38% had a disease of the cardiovascular system, and one-fifth (19%) had high cholesterol (e.g. hypercholesterolemia, hyperlipidaemia, raised triglycerides). Around 16% had COPD and 14% had some form of visual impairment. BMI data were available for 30 patients; the mean score for our sample was 31.7, classified as ‘obesity class one’ by the World Health Organisation (WHO). Nine patients met the threshold for ‘pre-obesity’, and 19 patients for obesity classes one, two or three. Only two patients were in the ‘normal weight’ range. On average, patients were prescribed 2.6 medications for regular use, with an average anticholinergic effect on cognition score of 5, according to the scoring system described in ( Bishara *et al.*, 2017).

The EQ-5D-5L domains mobility, self-care, usual activities, pain and discomfort, anxiety and depression were all significantly positively correlated with each other. They were all also negatively correlated with the broader EQ-5D-5L indicators of ‘health today’ and the overall ‘index value’ (a measure of overall wellbeing). Patients' assessment of their health on that specific day was linked with cognitive impairment scores ( *ρ*= -.432) whilst their overall health index score on the same questionnaire was not.

### Demographic characteristics and patient outcomes

Age was not significantly linked to any of the outcomes measured. Length of stay in current setting was positively associated with usual activities ( *ρ*= .440) and anxiety and depression ( *ρ*= .384) indicating a greater number of problems in these domains, and negatively associated with EQ-5D-5L index value suggesting poorer health ( *ρ*= -.374). Despite the correlation with the index value, there was no significant relationship with patients' assessments of their health on that specific day.

**Figure 1.**
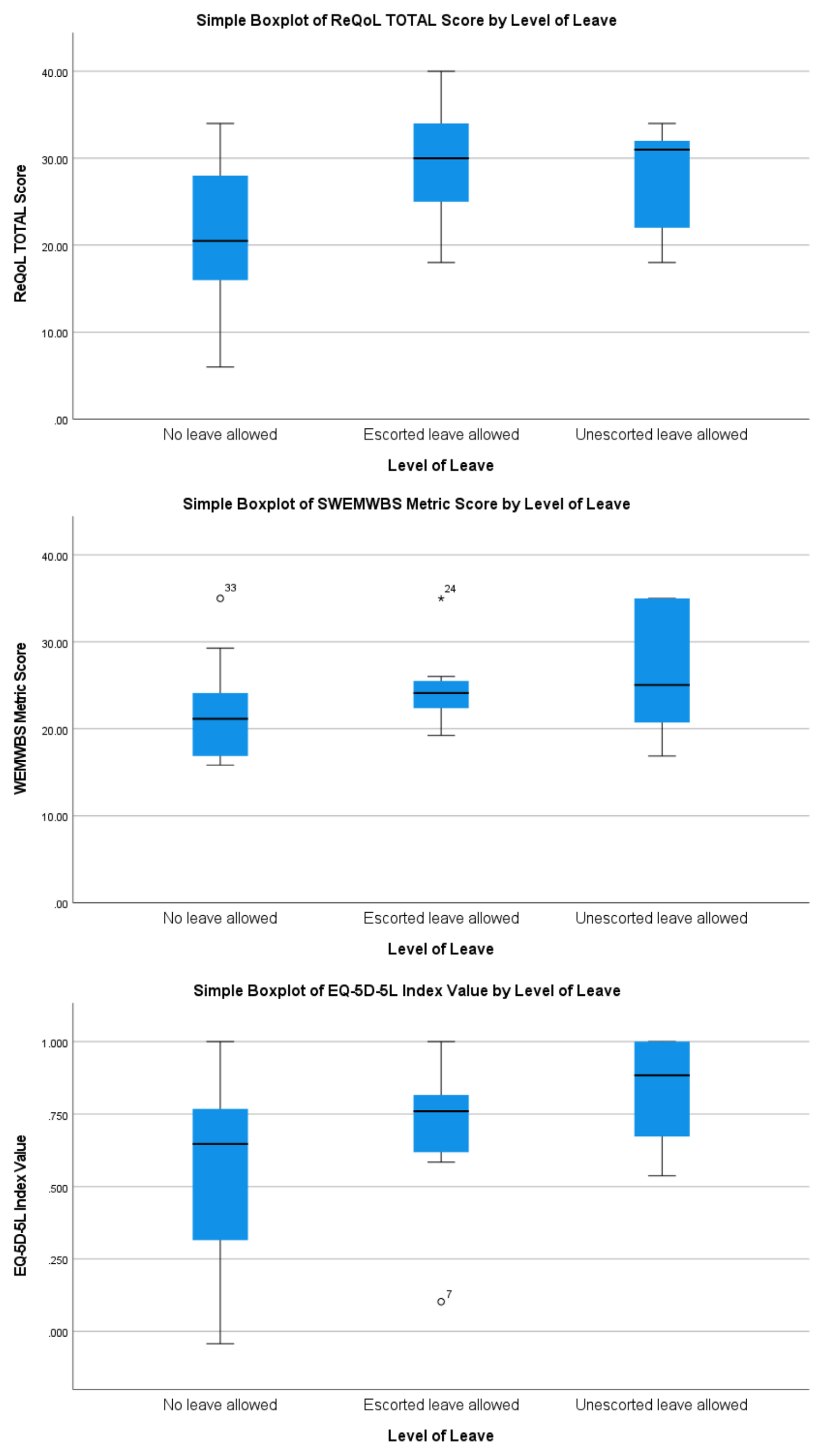
Mental health and wellbeing across levels of leave.

**Figure 2.**
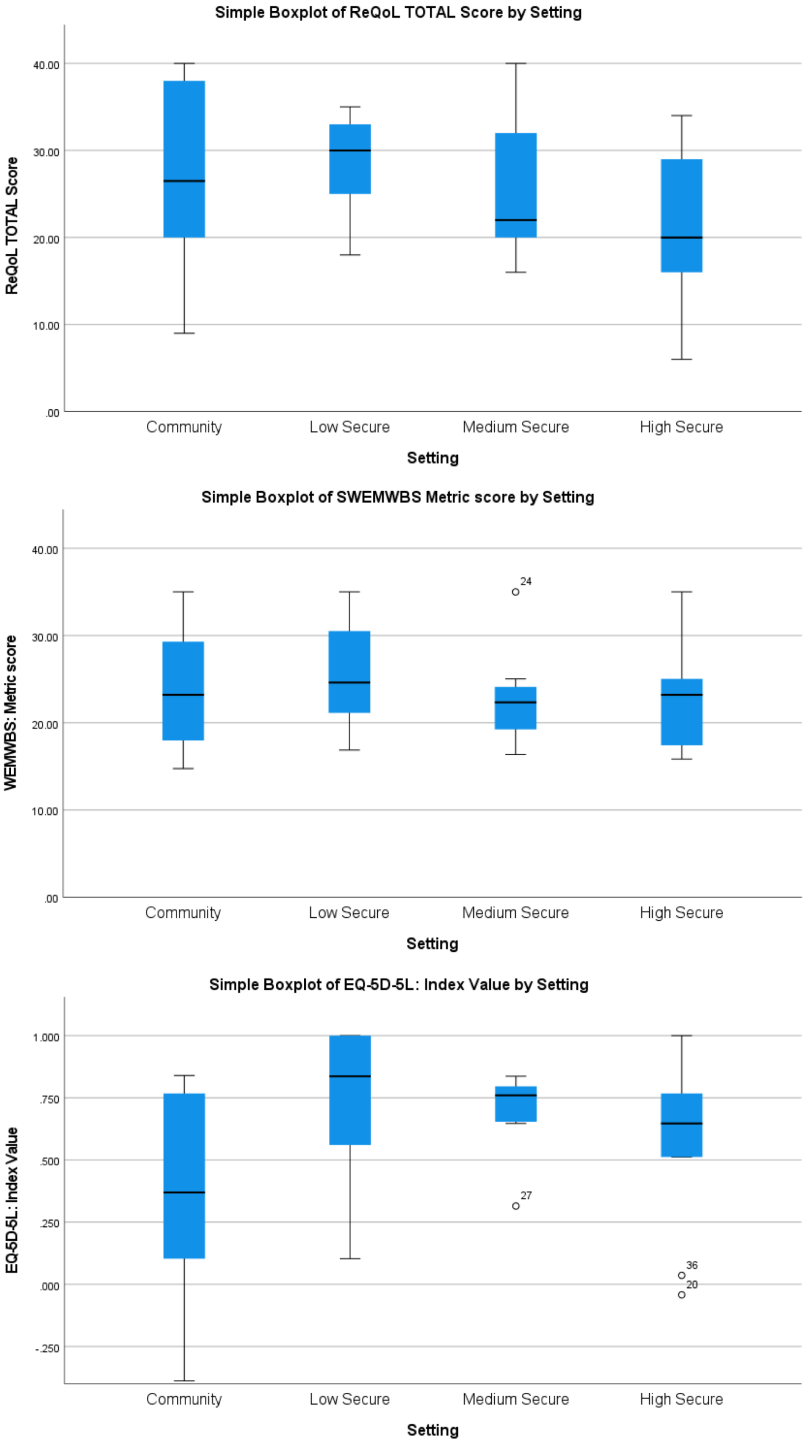
Mental health and wellbeing across treatment settings.

Mild cognitive impairment scores were negatively correlated with recovery-related quality of life scores ( *ρ*= -.377) and positively with higher restrictiveness ratings (for inpatients, *ρ*=.474). Premorbid IQ was negatively correlated with the EQ-5D-5L ‘health today’ domain ( *ρ*= -.432). As our sample was too small to conduct analyses of difference between more than two groups (i.e. ANOVA), Figure 1 and Figure 2 demonstrate a tentative finding that were ANOVAs to be conducted, significant differences would be unlikely on measures of recovery-related quality of life, overall wellbeing (EQ-5D-5L index value), mental wellbeing, or experiences of restrictiveness compared across levels of leave or treatment setting.

### Comparison with population norms and published samples

Compared to a sample of UK general mental health patients receiving care across different settings ( *M*=21.99; see Keetharuth & colleagues (2018) for more descriptive information), our sample reported significantly higher recovery-related quality of life scores on the ReQoL ( *M*=25.7) (t(35)=2.509, *p*=0.017, *d*=.418); when compared to a representative sample of the UK general population ( *M*=28.5) our sample did not differ significantly ( *t*(35)=-1.868, *p*=0.07). There was no significant difference between our sample ( *M*=23.5) and general population values ( *M*=23.6, see Stewart-Brown *et al.*, 2009) on the SWEMWBS measure of mental wellbeing ( *t*(35)=-0.107, *p*=0.915). Our sample ( *M*=32.9) reported similar scores on the measure of patient experiences of restrictiveness in secure care to the sample in Tomlin *et al.* (2019) ( *M*=35.6) ( *t*(26)=-.905, *p*=0.374). There was also no difference in relation to mild cognitive impairment measured with the MoCA between our sample ( *M*=23.5) and population norms for adults who have primary or no education ( *M*=23.5) ( *t*(33)=-0.045, *p*=0.965). Our sample scored significantly lower on the MoCA than adults living in Ireland with secondary education ( *M*=25.3) (t(33)=-2.773, *p*=0.009, *d*=-.475) and tertiary education ( *M*=26.7) ( *t*(33)=-4.894, *p*<.001, *d*=-.839) (see Kenny *et al.* (2013) for these values).

## Discussion

This article describes a sample of 37 forensic mental health patients aged 55 and over in forensic community and inpatient mental healthcare services. It makes a novel contribution to the literature by expanding the relative paucity of published data on this patient group and by investigating their physical health, health-related quality of life, recovery-related quality of life, mental wellbeing, experiences of restrictiveness in secure care **,**cognitive ability and demographics and comparing several of these outcomes to population norms or published data.

In some respects, our findings align with other cross-sectional studies of this group. Other studies also report: lower proportion of women patients than that reflected in the total forensic population ( Coid *et al.*, 2002; women are approximately 18% of the total forensic inpatient population in England and Wales, see Tomlin *et al.*, 2021); a high proportion of serious offences against the person (e.g. murder/manslaughter, assault) ( Di Lorito *et al.*, 2018b); multiple chronic physical health needs alongside complex mental health needs ( Girardi *et al.*, 2018b), and cognitive impairment and high rates of obesity as classified by BMI ( Di Lorito *et al.*, 2019).

On this last point, and considering cardiovascular health more broadly, a recent review of cardiometabolic disease in patients with psychosis of all ages in secure settings reported a weighted pooled prevalence of BMI scores >30 across eight studies of 39.8% (n= 1359); five studies of which reported a weighted pooled prevalence of BMI scores >25 at 72.4% (n= 840) ( Ma *et al.*, 2020). Weighted pooled prevalence scores were also reported for metabolic syndrome: 23.5% (k= 5; n= 1,390); diabetes: 11.3% (k= 12; n= 2,561); dyslipidaemia: 29.2% (k= 8; n= 1,135); hypertension: 25% (k=5; n= 857); cardiovascular disease: 15.6% (k=6; n= 1,047). Although direct comparison is not possible given different methods of diagnosis and recording practices, rates of dyslipidaemia were lower in our sample, but diabetes and cardiovascular disease were much higher. Cognisant of methodological caveats, this disparity might reflect a worsening of some forms of cardiometabolic health as patients age and spend more time in care.

The negative relationship between mild cognitive impairment scores and recovery-related quality of life was significant, whilst there was a trend towards significance with mental wellbeing. Cognitive ability scores were commensurate to a representative sample of adults living in Ireland with primary level or no education ( Kenny *et al.*, 2013). Using the MoCA threshold of 25/30 or below to indicate possible mild cognitive impairment ( Nasreddine, 2017), we found that 22/37 (65%) of our sample could have mild cognitive impairment. Di Lorito & colleagues (2019) found that 21% of their sample had ‘cognitive impairment’ as measured on the CAMCOG. Older forensic patients with poorer cognitive skills will likely need greater support both in hospital and in the community to achieve their recovery goals. A meta-analysis of 49 RCTs comparing interventions to improve global cognition in individuals with mild cognitive impairment found the following intervention types were significantly more effective than control conditions: cognition-based, physical exercise, combined cognition-based and physical exercise, and antioxidants ( Xu *et al.*, 2021). Cognitive Stimulation Therapy (CST) has been suggested in the literature as an example of a cognition-based intervention for this population that can be undertaken via computer or tablet and be facilitated with the support of carers and is not resource intensive ( Natarajan & Mulvana, 2017).

Recovery-related quality of life scores were associated with more positive perceptions of/satisfaction with ‘usual activities’ ^5^. This finding underscores the importance of meaningful and accessible activities in the recovery process. Secure inpatient settings have a limited range of activities and community patients can face age-, physical health- or forensic-related barriers to participation ( de Smet *et al.*, 2015). Activities that are available to inpatients have been described as childish or boring or repetitive by older patients in our study ( Walker *et al.*, 2022b) and elsewhere ( Di Lorito *et al.*, 2018a; Visser *et al.*, 2021). Patients in the community also describe a paucity of appropriate and engaging activities ( de Smet *et al.*, 2015). Novel, patient-led, and health and age needs-appropriate activities for older patients should be a priority and can be inexpensive to implement compared to complex psychosocial or pharmacological interventions. 

Comparing our sample to population norms or published study data, this study found that older forensic patients subjectively rate their mental wellbeing at a level that is not significantly different from the general population ( Stewart-Brown *et al.*, 2009), and their recovery-related quality of life as not significantly different from the general population and better than adults receiving general mental healthcare ( Keetharuth *et al.*, 2018). Although forensic patients have complex comorbid physical and mental health needs, the level of healthcare assessment, monitoring and support they receive is likely to contribute to explaining the lack of significant differences from the general population on these measures. Qualitative studies of patients’ experiences of care suggest that this group likely has better access to healthcare, professional support, social contact, structured activities, regular food, and exercise equipment than the same age group in the community in the general population ( di Lorito *et al.*, 2018a; di Lorito *et al.*, 2017; Visser *et al.*, 2021; Walker *et al.*, 2022b). In line with this, mental wellbeing did not correlate with the ‘mobility’, ‘self-care’, and ‘pain/discomfort’ domains of the EQ-5D-5L.

The link between physical health and mental health was not entirely clear though, as the ‘anxiety and depression’ domain of the EQ-5D-5L did correlate with these three domains, and the ReQoL correlated with the ‘mobility’ EQ-5D-5L domain. This could mean that perceptions of ‘depression and anxiety’ are associated with physical health in a way the broader construct of mental wellbeing is not, or that this reflects a response bias given that these domains were all measured on the same questionnaire (the EQ-5D-5L). Nevertheless, given this and the link between mobility and recovery-related quality of life, the associations between physical health and general mental health should be investigated further and services should ensure barriers to mobility are removed.

Age was unrelated to the outcomes measured in this study. This might be because all our patients were 55 years and older (maximum of 70 years), offering little variance for the statistical analysis. One possible explanation for this null finding is that several qualitative investigations have found age to be a subjective construct for many forensic mental health patients; some reject the ‘older’ label and express *feeling* young ( Visser *et al.*, 2021). As our outcomes were largely subjectively measured it is possible some respondents *felt* younger or older than others the same age and rated their mental wellbeing, physical health and recovery-related quality of life in line with this. A larger sample with patients aged 18 and older might have led to different findings. Indeed, studies using staff-rated instruments have found that older patients were less likely to have healthcare needs met ( Das *et al.*, 2012); and were less likely to improve over the course of treatment on measures of security needs, self-harm, harm to others, mental health disturbance, personal wellbeing, emotional wellbeing, and socio-economic status ( Girardi *et al.*, 2018).

In relation to medication, the mean number of psychotropic drugs prescribed (2.1 per patient) does not seem excessive given the range and number of diagnoses. The higher total number of drugs (mean = 7.6 per patient) doubtless reflects the burden of physical morbidity, especially diabetes and cardiovascular conditions, in the sample. Of concern is that the mean anticholinergic effect score was 2.4, which suggest that there is scope for review of these medications as they are known to contribute to the future risk of dementia ( Coupland *et al.*, 2019) and are associated with higher risk of mortality and emergency hospitalisation in people with established cognitive impairment ( Bishara *et al.*, 2020).

### Clinical and research implications

To summarise the practical implications of our study, our data suggest that older patients could benefit from interventions to improve cognition or ameliorate cognitive decline. Studies suggest this is best achieved through cognition-based interventions, physical exercise and antioxidants ( Xu *et al.*, 2021). Patient recovery-related quality of life and mental wellbeing is likely enhanced by engagement in a range of meaningful and age- or needs-appropriate activities that include work, study, housework, family or leisure activities. Services should continue to address physical healthcare needs, especially relating to cardiovascular health, as patients progress into the community to ensure that physical health concerns do not hinder mental wellbeing and recovery.

The negative correlation of recovery-related quality of life with problems related to depression and anxiety, ability to perform usual activities (work, study, housework, family or leisure) and mobility highlights the importance of seeking to address these issues to enhance quality of life. To reduce levels of obesity and diabetes, more consideration should be given to improving patient physical activity levels, diet, and sleep quality. Acknowledging that many patients will be experiencing cognitive impairment, services should make allowances for this in provision of services, needs assessment, risk assessment, interventions, and treatment, as well as providing relevant staff training.

Further research should address participation in meaningful activities in more detail. Tools that measure aspects of engagement in occupational activities such as the Model of Human Occupation Screening Tool (MOHOST; see: Fan *et al.*, 2016) or the Engagement in Meaningful Activity Survey (EMAS; see: O’ Flynn *et al.*, 2018) should be used. These studies should be longitudinal and compare age groups. This would complement the growing literature assessing levels of met and unmet need in this population ( Girardi *et al.*, 2018). Other research could evaluate CST-based interventions, potentially delivered via iPad app, in this group.

### Limitations

Our study has some limitations. Our sample size can be considered relatively small ( *N*=37), precluding the use of multivariate analysis (e.g. regression) or comparisons of mean differences across more than two groups (e.g. ANOVA). However, our sample is sufficiently powered for the correlations and t-tests conducted. Further, our sample size is comparable to other studies of this population ( Coid *et al.*, 2002; Das *et al.*, 2011; Das *et al.*, 2012; Di Lorito *et al.*, 2019; Girardi *et al.*, 2018; Lightbody *et al.*, 2010; Tomar *et al.*, 2005), most of which are retrospective using hospital records and did not involve active participant recruitment. The internal consistency of the MoCA was questionable (α=.67), falling just below the generally accepted α=.70 ( Bland & Altman, 1997). Thus, conclusions concerning mild cognitive impairment in this study should be read with some caution. Nonetheless, our findings in this regard are similar to other studies ( Di Lorito *et al.*, 2019).

## Conclusion

In consultation with a lived experience advisory panel to identify our most important research foci, we investigated correlates of patients’ health-related quality of life, recovery-related quality of life and mental wellbeing. We found that recovery-related quality of life was significantly associated with a measure of mild cognitive impairment and problems engaging in usual activities. Mental wellbeing was trending towards a significant relationship with problems engaging in usual activities ( *p*=0.54). Perceptions of physical health were largely though not entirely uncorrelated to either of these constructs (the exception being recovery-related quality of life and mobility). Age was not correlated with health-related quality of life, recovery-related quality of life or mental wellbeing. There were high levels of possible mild cognitive impairment. Diabetes, vitamin D deficiency, and musculoskeletal and cardiovascular conditions were prevalent. We suggest services co-develop with patients age-appropriate meaningful activities that are mindful of mobility issues and consider implementing cognitive stimulation therapies. This study adds to the growing and much needed literature on older forensic mental health patients and further promotes the importance of studying different marginalised patient groups.

## Data availability

### Underlying data

Open Science Framework: Older adult forensic mental health patients: defining needs, barriers, facilitators and 'what works' to enable better quality of life, health and wellbeing and to reduce risk, https://doi.org/10.17605/OSF.IO/GS37Y. ( Tomlin *et al.,* 2022)

This project contains the following underlying data:

- 2021.11.07 ENHANCE Data presented in Older forensic mental healthcare patients in England- Demographics, physical health, mental wellbeing, cognitive ability and quality of life.sav

### Extended data

Open Science Framework: Older adult forensic mental health patients: defining needs, barriers, facilitators and 'what works' to enable better quality of life, health and wellbeing and to reduce risk, https://doi.org/10.17605/OSF.IO/GS37Y. ( Tomlin *et al.,* 2022)

This project contains the following extended data:

- 2022.01.19 Tomlin *et al.* 2022 Correlations Table.pdf

Data are available under the terms of the Creative Commons Attribution 4.0 International license (CC-BY 4.0).
