# Older forensic mental healthcare patients in England: demographics, physical health, mental wellbeing, cognitive ability and quality of life

**DOI:** 10.3310/nihropenres.13248.2

**Published:** 2022-06-22

**Authors:** Jack Tomlin, Kate Walker, Jen Yates, Tom Dening, Birgit Völlm, Chris Griffiths

**Affiliations:** 1 School of Law and Criminology, University of Greenwich, London, UK; 2 Innovation and Research Department, Northamptonshire Healthcare NHS Foundation Trust, Northampton, UK; 3 Division of Psychiatry and Applied Psychology, School of Medicine, University of Nottingham, Nottingham, UK; 4 Department of Forensic Psychiatry, University Hospital Rostock, Rostock, Germany

**Keywords:** Forensic mental health, older patients, quality of health, mental wellbeing, recovery

## Abstract

**Background:**

Older individuals (e.g., 55 years and over) constitute a growing proportion of the forensic mental health patient population. As a group, they are vulnerable to health outcomes similar to other individuals with serious mental disorders of the same age; however, these concerns can be compounded by complex forensic-related care backgrounds and clinical presentations, lengthy periods of time spent in prison or psychiatric hospitals, substance use histories, and crime perpetration or victimisation. The healthcare needs and strengths of this group are not well understood.

The aim of this study was to identify and describe the demographic, physical health, mental wellbeing, cognitive ability, and quality of life profiles of older forensic patients in community, low, medium, and high security settings in England.

**Methods:**

A cross-sectional quantitative study design was used. N=37 forensic patients aged 55 years and over completed six questionnaires. Data were also collected from patient records.

**Results:**

Most patients were male and were diagnosed with psychosis. The most frequently committed index offence types were violent offences. Patients were prescribed 7.6 medications on average and had average anticholinergic effect on cognition scores of 2.4. Nearly half the sample had diabetes, with an average BMI score of 31.7 (indicating obesity). Possible cognitive impairment was identified in 65% of the sample. Patients’ assessments of their recovery-related quality of life and mental wellbeing were comparable to published UK general population values. Assessments of quality of life were positively correlated with the ability to undertake everyday activities and cognitive performance.

**Conclusions:**

We suggest that forensic services are well-placed to provide holistic mental and physical care to this group but that they should co-develop with patients a greater range of age-appropriate meaningful activities that are mindful of mobility issues and consider implementing more cognition-based and physical health interventions.

## Plain language summary

Forensic mental health services provide care for people who have committed a crime or are at risk of harm to themselves or others. This care takes many forms, including mental health support, physical health care, and assistance to re-enter the community after spending time in secure mental health hospitals. Due to changes in the general population, there is a growing number of patients over the age of 55 in care. Despite this, we do not know enough about the healthcare needs of this older patient group and how these needs differ from younger patients. This study aimed to find out more about this patient group.

In the current study, thirty-seven forensic patients aged 55 years and older were recruited from low, medium and high security hospitals and community care. These patients completed six questionnaires. Patients were asked about their physical health, mental wellbeing, cognitive ability, and quality of life. Information about medical diagnoses and socio-demographic backgrounds were collected from hospital records.

The study found that these patients had complex health needs. Most patients were diagnosed with psychosis (e.g., schizophrenia), were men, and had committed a violent criminal offence. On average patients were prescribed 7.6 medications, many of which carry significant side effects. Nearly half of the patients had diabetes, and most patients were obese or overweight. Quality of life scores were lower for patients who also reported having problems undertaking everyday activities and patients with cognitive impairment. Patients subjectively rated their mental wellbeing and quality of life similarly to the general population’s ratings as reported in other studies. We suggest that services should work with patients to develop a greater number of age-appropriate activities and interventions aimed at improving cognitive and physical health.

## Introduction

Providing support, care and treatment for forensic patients that is responsive to their individual needs and strengths is a core tenet of the recovery approach ( Simpson & Penney, 2018). Recognising this requires investigating the individual profiles of patients in forensic mental health services and how patients’ backgrounds, characteristics, experiences and perspectives differ. This approach can be seen in recent efforts to develop services and interventions responsive to the lived experiences, strengths and needs of, amongst others, women patients ( de Vogel & Nicholls, 2016), culturally and ethnically diverse patients ( Hui, 2017), Deaf patients ( Wakeland *et al.*, 2019), and older patients ( Solares *et al.*, 2020). This latter group was the focus of the ENHANCE study ^1^, which investigated the demographics, health-related quality of life, recovery rated quality of life, mental wellbeing, cognitive ability, wellbeing and secure hospital restrictiveness rating profiles of forensic mental healthcare patients aged 55 and over.

Forensic mental healthcare patients aged 50 and over constitute about 20% of the UK forensic mental healthcare inpatient population ( Di Lorito *et al.*, 2019; Di Lorito *et al.*, 2018b). This proportion is likely to increase as the population ages ( World Health Organization, 2017); indeed, the number of forensic inpatients over 65 in Scotland increased by 50% and those aged 56–65 by 27% between 2013 and 2019 ( Scottish Government, 2021). The physical and mental healthcare needs of this group are complex as often comorbidities are prevalent ( Natarajan & Mulvana, 2017). Many forensic mental healthcare patients have biographies characterised by placements in psychiatric institutions and prisons, and have experiences of substance abuse and long-term serious mental and physical illnesses for which they may have not received appropriate treatment or management ( Centre for Mental Health, 2010; Centre for Mental Health, 2013). This constellation of factors means that many forensic patients experience an ‘accelerated aging’, presenting with a level of health need at, for example, 50 years old that would be equivalent to an average member of the general public at 60 years ( Merkt *et al.*, 2020).

Compared to younger adult mental healthcare patients, older individuals are more likely to have a higher number of unmet health needs and to experience fewer improvements in their health over time ( Das *et al.*, 2011; Das *et al.*, 2012; Girardi *et al.*, 2018). Older patients are more likely to be diagnosed with depression, organic brain syndrome, or delusional disorder ( Coid *et al.*, 2002). Disabling health issues are more prevalent in older patients; these include cognitive decline, mobility problems and sensory impairment ( Di Lorito *et al.*, 2019). The number of medications given to older patients has been found to double throughout placement in secure hospitals, highlighting a decline in health ( Lightbody *et al.*, 2010). Investigating and documenting the disparities between younger and older mental healthcare patients can better equip us to shape service provision, co-develop responsive and appropriate interventions with patients, and address structural disparities in health and wellbeing outcomes ( Hui *et al.*, 2021).

## Aims and rationale of this study

Despite the growing research and clinical interest in older adult forensic mental health patients there remains a paucity of research data. The current study describes the quality of life, physical health, mental wellbeing, cognitive ability, demographics, and experiences of restrictiveness in secure care of 37 forensic mental healthcare patients, aged 55 and older, from community and low, medium and high security inpatient settings in England.

The research aims were co-authored with the ENHANCE study’s Lived Experience Advisory Panel (LEAP) (a group made up of people with lived experience of mental health issues and forensic mental healthcare service use). These aims were to investigate whether physical health, health-related quality of life, and recovery-related quality of life were correlated with each other in this population, and whether these constructs were associated with: mild cognitive impairment, age, length of stay in secure care, amount of leave (for inpatients), experiences of secure hospital restrictiveness, and treatment setting (i.e. community or low, medium or high security in-patient hospital). 

## Methods

### Sampling and recruitment

A stratified cluster sampling frame was planned for community, low and medium secure units, taking into account gender mix and specialisation (e.g. patients with personality disorders or intellectual disabilities). This was disrupted due to COVID-19; however, we were able to recruit participants from a variety of settings across a geographically diverse range of sites. NHS Trusts were recruited through the Clinical Research Network (CRN). No specialist facilities were recruited, sites provided low, medium, and or high secure facilities and community care. Of the 12 community patients recruited, 11 were living independently, with one living in supported accommodation. 

Local investigators liaised with members of the study team to identify patients aged over 55 years. These patients were then approached by local investigators to ascertain interest in participation, provide information sheets, and answer any questions about the study. Inclusion criteria for patients was those: aged 55 or over; under the care of forensic mental health services; able to complete self-report questionnaires and semi-structured interviews; who understood written and oral English; and who had capacity to consent. The CRN and PIs at different sites undertook recruitment and initial consenting for participation and as a result it was unknown how patients were approached and how many refused. The number of participants recruited from each trust is depicted in Table 1.

**Table 1.** Number of recruits by participating NHS trusts.

	Total number of recruits	Security levels
Trust 1	4	C = 4
Trust 2	4	MS = 3 LS = 1
Trust 3	4 (3 included, 1 excluded)	MS = 1 LS = 3 (1 excluded)
Trust 4	14	HS = 8 MS = 4 C = 2
Trust 5	4	LS = 2 C = 2
Trust 6	4	LS = 2 C = 2
Trust 7	2	C = 2
Trust 8	2	MS = 1 LS = 1

Notes: C = community; L = low security; MS = medium security; HS = high security

55 years was chosen as the cut-off as this reflects the expedited ageing experienced by forensic patients, suggested by some to be around 10 years ( Merkt *et al.*, 2020), which aligns with an often-used older age threshold in non-forensic populations used in research of 65 years. The ENHANCE study also included interviews with staff members from these services (reported separately) and patients known to these professionals were invited to take part. All participants were able to provide informed consent to complete questionnaires and to be interviewed. A sample size of N=36 was sought as this was considered sufficient to achieve saturated themes in the qualitative aspects of this project (reported elsewhere) and post-hoc power analyses report the obtained power of the correlations investigated in this study (see ‘Data analysis’).

### Ethical approval

Ethical approval was granted by the NHS Health Research Authority (IRAS: 258016; REC: 19/EM/0350). Funding was provided by the National Institute for Health Research [PB-PG-1217-20028].

### Data collection

Data collection took place between March 2020 and September 2021 across eight National Health Service (NHS) trusts. In total, 38 patients were recruited and completed all study questionnaires. However, it transpired one patient was 53 years old, so their data are excluded from the analysis. Meetings between the researcher responsible for data collection and participants took place in person ( *n*=10), via video call ( *n*=26), or over the phone ( *n*=1). All patients gave informed consent, with written consent taken from those met face-to-face, and verbal recorded consent from those met via video call or phone. Both methods of recording consent were approved by the relevant ethics committee.

Clinical, legal and demographic data ( Tomlin *et al.*, 2022) were extracted from patient clinical records by principal investigators based at each recruitment site. Legal data included length of stay in the service, nature of the index offence(s) and Mental Health Act 1983 status. Index offences were categorised according to the UK Home Office Offence Classification Index. Where a patient had more than one index offence, we report the most severe as indicated by the Home Office Crime Severity Score. It should be noted that in England and Wales, patients do not need to have committed an index offence to receive treatment in forensic services. They might receive treatment in these services under a civil, non-forensic legal section where they are at risk of harm to themselves or others, which cannot be safely managed in general psychiatric settings.

Clinical data included ICD-10 diagnoses, body mass index (BMI), lists of physical health conditions and medication data (total number of drugs currently prescribed, number of psychotropic drugs currently prescribed, and Anticholinergic Effects on Cognition scores ( Bishara *et al.*, 2017)). Medications were included if prescribed for regular consumption. As required ( *pro re nata* or prn) prescriptions were not counted as it would not be possible to ascertain how much of the drug had actually been administered.

Patients completed six questionnaires:

Q1. The Short Warwick-Edinburgh Mental Wellbeing Scale (SWEMWBS; Tennant *et al.*, 2007). This is an overall mental wellbeing measure, consisting of seven self-report questions and Likert scale responses. Higher total ‘metric scores’ indicate better mental wellbeing. Metric scores range from 7 to 35. A score of 18 or less is indicative of probable clinical depression and scores of >18–20 are indicative of possible mild depression.
Q2. The EQ-5D-5L ( Devlin *et al.*, 2018) is a measure of overall health-related quality of life. It has one self-report question asking for a ‘health today’ score (a higher score indicates better health), and five self-report questions targeting the domains: ‘mobility’, ‘self-care’, ‘usual activities’, ‘pain and discomfort’, and ‘anxiety and depression’ (higher scores on these domains indicate a greater number of problems). Likert scale responses are used. Researchers calculate an ‘index score’, which summarises responses across these five domains. Index scores range from just under 0 to 1, with scores under 0 indicating health states equivalent to or worse than death and 1 suggesting good health ( Devlin *et al.*, 2018).
Q3. The Recovering Quality of Life measure (ReQoL-10; Keetharuth *et al.*, 2018) is an overall recovery-related quality of life questionnaire. This has 10 self-report questions with Likert scale responses. Higher scores indicate more positive quality of life. Scores range from 0 to 40, with scores up to 24 representing the clinical range and scores 25 and greater reflecting the general population.
Q4. The Cambridge Contextual Reading Test – Short Version (Short CCRT; Beardsall, 1998). This is a reading task measure of premorbid IQ, wherein respondents must read sentences aloud that include difficult to pronounce words. Scores are calculated by noting the number of incorrectly pronounced words, such as ‘bouquet’, ‘thyme’ or ‘subtle’. Higher scores indicate better performance. Scores range from 0 to 25.
Q5. The Montreal Cognitive Assessment (MoCA; Nasreddine *et al.*, 2005) is a measure of cognitive impairment. It captures respondents’ performance across the domains: attention and concentration, executive functions, memory, language, visuoconstructional skills, conceptual thinking, calculations, and orientation. A total score out of 30 can be calculated, with higher scores indicating better performance. A score <26 indicates possible mild cognitive impairment.
Q6. The Forensic Restrictiveness Questionnaire (FRQ; Tomlin *et al.*, 2019). This measures inpatients’ experiences of the restrictiveness of secure inpatient care across 15, self-report Likert scale items. Higher scores indicate greater levels of perceived restrictiveness. Scores range from 15 to 75.

Due to disruptions due to COVID-19, our data collection methods had to be revised during data collection. Two sets of data were collected in person, where the participants completed the four self-report questionnaires (Q1 EQ-5D-5L; Q2 ReQol; Q3 SWEMWBS; Q4 FRQ), and the researcher undertook the two (Q5 MoCA; and Q6 CCRT) other questionnaires with the participants (Q5 and Q6 both require the researcher to actively administer them). The rest of the questionnaires were collected over video-call. For this, the researcher administered Q5 and Q6 to the participants, but Q1–Q4 were filled in by the participants independently to the call. Q1–Q4 were distributed to the participants by principal investigators on each site and were posted/emailed back to the research team once completed.

### Data analysis

Post-hoc power analyses were conducted as the number of completed questionnaires varied (e.g., SWEMWBS *n* =36, and FRQ *n* =27). G*Power ( Faul *et al.*, 2007) suggested that at *r* = 0.5, *p* = 0.05, two-tailed, n = 36, our analysis yielded a sufficient power of 0.89. G*Power also indicated at *r* = 0.5, *p* = 0.05, two-tailed, n = 27, that analyses including the FRQ yielded a power of 0.78, just under the usually accepted standard of 0.8 (in this study achieved with a sample of *n* =28). This suggests that whilst most correlations conducted in this study are sufficiently powered, our findings involving the FRQ should be seen as exploratory.

Three percent of the questionnaire data were missing, largely due to nine patients not completing the Short CCRT, reporting difficulties with eyesight, reading level or not wanting to complete this. Eight percent of the demographic, legal and clinical data were missing due to recording issues in patient files. Pairwise deletion was used to handle missing data. IBM’s statistical package for the social sciences (SPSS) software v.27 was used. The distribution of questionnaire response data was assessed with the Shapiro-Wilk statistic and most variables were non-normally distributed. To account for this, non-parametric methods were used.

The internal consistency of the questionnaires used in the study was assessed prior to analysis. This suggested all questionnaires were appropriate to use: SWEMWBS, α= .886, *n*=36; EQ-5D-5L, α= .871, *n*=36; ReQoL, α= .859, *n*=36; FRQ, α= .945, *n*=27; and MoCA, α= .660, *n*=34. The alpha value (α) for the MoCA was lower than for the other measures and much of the literature ( Nasreddine *et al.*, 2005) but other studies ( Bernstein *et al.*, 2011) have reported values lower than α=.7 (the recommended cut-off for assuming adequate internal consistency; Bland & Altman, 1997). Spearman’s RHO ( *ρ*) was used to assess correlations ^2^. Effect sizes ( *r*) were judges as follows: 0.1, 0.3, and 0.5 as small, medium and large ( Cohen, 1988). Statistical significance was set at *p*=<0.05; effect sizes are reported where appropriate. All correlation coefficients reported without a *p* value are significant at *p*=<0.001.

## Results

### Descriptive statistics

Table 2 presents the demographic, clinical and legal profiles of the patient group. Table 3 and Table 4 give an overview of the mental health and physical diagnoses given to patients in the sample. These show that participants were mostly men (92%), of white British ethnicity (81%), with a mean age of 60 years. Median length of stay in current institution was 1404 days (approximately 45 months). The most frequently diagnosed mental disorders were schizophrenia, schizotypal, and delusional disorders (60%), any type of personality disorder (41%), and then mental and behavioural disorders due to psychoactive substance use and mood (affective) disorders (both at 16.2%) ^3^. Scores for each questionnaire are presented in Table 5.

**Table 2.** Demographic, clinical and legal characteristics of the sample.

Characteristic	Frequency / mean / median	% / SD / 25th & 75th
**Age** ( *n*=37)	*Mn*= 59.8	*SD*= 3.9
**Sex** ( *n*=37)		
- Men	34	92
- Women	3	8
**Ethnicity** ( *n*=37)		
- White	30	81
- Black, African, Caribbean, or Black British	6	16
- Mixed or multiple ethnic group	1	3
**Mental Health Act 1983 section** ( *n*=37)		
- No legal section (community treatment)	5	14
- s. 3 (civil admission for treatment)	3	8
- s. 37/41 (hospital order and restriction order)	11	30
- s. 37/42 (hospital order and lifted restriction order)	1	3
- s. 41 (treatment in the community and restriction order)	2	5
- s. 41 (5) (notional hospital order)	2	5
- s. 42 (treatment in the community and lifted restriction order)	2	5
- s. 45 (A) (hybrid treatment order)	1	3
- s. 47/49 (prison transfer and restriction order)	9	24
- s. 117 (aftercare following discharge)	1	3
**Index offence** ( *n*=37)		
- (Attempted) Murder / Manslaughter	11	30
- Violence against the person	8	21
- Sexual offences	8	21
- Robbery	2	5
- Possession of weapons	1	3
- Threatening to destroy or damage property	1	3
- No offence	6	16
**Setting** ( *n*=37)		
- Community	10	27
- Low secure	8	22
- Medium secure	9	24
- High secure	10	27
**Length of stay in days** ( *n*=32)	*Mdn*= 1404	25 ^th^= 469; 75 ^th^= 3803
**Number of current prescribed medications** ( *n*=37)	*Mn*= 7.6	*SD*= 4.4
**Number of current prescribed psychotropic medications** ( *n*=37)	*Mn*= 2.1	*SD* = 1.5
**Anticholinergic effect of medications on cognition scores** ( *n*=37)	*Mn*= 2.4	*SD* = 2.1
**Body Mass Index** (BMI; *n*=30)	*Mn*= 31.7	*SD* = 4.5
**Possible** **mild cognitive impairment according to MoCA** ( *n*=34)		
- Yes	22	65
- No	12	35

Notes. Percentages of observed values, i-e- excluding missing values. *Mdn*, median; *Mn*, mean; *SD*, standard deviation; 25 ^th^ and 75 ^th^ percentiles.

**Table 3.** Mental health diagnoses in the sample.

Diagnoses ordered by ICD-10 categories	Frequency	%
Organic, including symptomatic, mental disorders	1	2.7
Mental and behavioural disorders due to psychoactive substance use	5	13.5
Schizophrenia, schizotypal and delusional disorders	22	59.5
Mood [affective] disorders	6	16.2
Neurotic, stress-related and somatoform disorders	3	8.1
Personality disorders (Any)	15	40.5
- Dissocial	5	13.5
- Dependent	3	8.1
- Avoidant (anxious)	5	13.5
- Emotionally Unstable	4	10.8
- Paranoid	4	10.8
- Schizoid	2	5.4
- Antisocial	4	10.8
- Borderline	3	8.1
- Mixed Personality Disorder	2	5.4
Disorders of sexual preference	1	2.7
Disorders of psychological development	2	5.4

Notes. Observations greater than 37 and percentages greater than 100 as most patients had multiple diagnoses. *N*=37.

**Table 4.** Physical health burden of the sample.

Physical diagnoses	Frequency	%
Diabetes	18	48.7
Cardiovascular and circulatory system	14	37.8
High cholesterol (e.g. hypercholesterolemia, hyperlipidaemia, raised triglycerides)	7	18.9
Chronic obstructive pulmonary disease (COPD)	6	16.2
Visual impairment	5	13.5
Asthma	4	10.8
Vitamin D deficiency	4	10.8
Diseases of the musculoskeletal system and connective tissue	3	8.1
Hearing loss	1	2.7
Impaired Physical Mobility	1	2.7

Notes. *N*=37.

**Table 5.** Questionnaire scores of the sample.

Questionnaire	Mean	SD
SWEMWBS Metric Score ( *n*=36)	23.5	6.3
ReQoL Total Score ( *n*=36)	25.7	8.9
EQ-5D-5L Index Value ( *n*=36)	0.6	0.4
FRQ Total Score ( *n*=27)	32.9	15.7
MoCa Total Score ( *n*=34)	23.5	3.8
Short CCRT ( *n*=28)	19.3	4.7

### Mental wellbeing

Mental wellbeing (SWEMWBS) was significantly positively associated with recovery-related quality of life (ReQoL) ( *ρ*= .773), and ‘health today’ ( *ρ*= .486) ^4^ as assessed by the EQ-5D-5L, both considered at or above large effect sizes. It was negatively correlated with the depression and anxiety domain ( *ρ*= -.348) of the EQ-5D-5L and was trending towards a significant relationship with the ‘usual activities’ domain of the same measure (meaning fewer problems in these domains; *ρ*= -.324, p=.054). A negative correlation was also observed for inpatient perceptions of restrictiveness in care ( *ρ*= -.481)), a large effect but the small sample size must be borne in mind here. The association between mental wellbeing and mild cognitive impairment was trending towards significance in a negative direction ( *ρ*= -.320, *p*=.065).

### Recovery-related quality of life

Recovery-related quality of life positively correlated with the EQ-5D-5L ‘health today’ domain ( *ρ*= .627), an above large effect size, and its overall index value ( *ρ*= .362). Recovery-related quality of life was negatively correlated with ‘mobility’ ( *ρ*= -.340), ‘usual activities’ ( *ρ*= -.551), ‘depression and anxiety’ domains on the EQ-5D-5L ( *ρ*= -.408) implying fewer problems on these domains, and (for inpatients) experiences of restrictiveness ( *ρ*= -.608), a large effect but the small sample size must be borne in mind here. The association between recovery-related quality of life and mild cognitive impairment was also significant in a negative direction ( *ρ*= -.377).

### Health status and perceptions of physical wellbeing

Nearly half (49%) our sample were diagnosed with diabetes, 38% had a disease of the cardiovascular system, and one-fifth (19%) had high cholesterol (e.g. hypercholesterolemia, hyperlipidaemia, raised triglycerides). Around 16% had COPD and 14% had some form of visual impairment. BMI data were available for 30 patients; the mean score for our sample was 31.7, classified as ‘obesity class one’ by the World Health Organisation (WHO). Nine patients met the threshold for ‘pre-obesity’, and 19 patients for obesity classes one, two or three. Only two patients were in the ‘normal weight’ range. On average, patients were prescribed 2.1 psychotropic medications for regular use, with an average anticholinergic effect on cognition score of 2.4, according to the scoring system described in ( Bishara *et al.*, 2017).

The EQ-5D-5L domains mobility, self-care, usual activities, pain and discomfort, anxiety and depression were all significantly positively correlated with each other. They were all also negatively correlated with the broader EQ-5D-5L indicators of ‘health today’ and the overall ‘index value’ (a measure of overall wellbeing). Patients' assessment of their health on that specific day was linked with cognitive impairment scores ( *ρ*= -.432) whilst their overall health index score on the same questionnaire was not.

### Demographic characteristics and patient outcomes

Age was not significantly linked to any of the outcomes measured. Length of stay in current setting was positively associated with usual activities ( *ρ*= .440) and anxiety and depression ( *ρ*= .384) indicating a greater number of problems in these domains, and negatively associated with EQ-5D-5L index value suggesting poorer health ( *ρ*= -.374). Despite the correlation with the index value, there was no significant relationship with patients' assessments of their health on that specific day.

Mild cognitive impairment scores were negatively correlated with recovery-related quality of life scores ( *ρ*= -.377) and positively with higher restrictiveness ratings (for inpatients, *ρ*=.474). Premorbid IQ was negatively correlated with the EQ-5D-5L ‘health today’ domain ( *ρ*= -.432). As our sample was too small to conduct analyses of difference between more than two groups (i.e., ANOVA), we present median recovery-related quality of life, overall wellbeing (EQ-5D-5L index value), mental wellbeing, and experiences of restrictiveness scores across treatment settings and levels of leave in Table 6. Though it is not possible to draw firm conclusions from these findings, at face value there appears to be a trend indicating that scores improve as levels of security decrease from high to low and as levels of leave increase. The exception to this is that community patients appear to have equivalent or poorer outcomes on the SWEMWBS and EQ-5D-5L than inpatients, and ReQoL scores at a level between patients in medium and low security.

**Table 6.** Mental health and wellbeing across treatment settings and levels of leave.

	ReQoL	SWEMWBS Metric	EQ-5D-5L Index	FRQ
Level of Leave				
None	20.5 (12.25)	21.165 (7.59)	.647 (.523)	32.5 (23.5)
Escorted	30 (13)	24.11 (4.48)	.760 (.253)	31 (24)
Unescorted	31 (11.5)	25.065 (15.23)	.884 (.361)	23.5 (42.5)
Setting				
Community	26.5 (18.25)	23.21 (12.43)	.429 (.698)	-
Low	30 (10)	24.62 (11.82)	.836 (.451)	27.5 (36)
Medium	22 (13.5)	22.35 (6.51)	.760 (.166)	30 (11)
High	20 (18)	23.21 (10.28)	.647 (.610)	36 (38.5)

Notes: Median values (interquartile ranges). No FRQ values reported for community patients as the FRQ is a measure of inpatient experience.

## Discussion

This article describes a sample of 37 forensic mental health patients aged 55 and over in forensic community and inpatient mental healthcare services. It makes a novel contribution to the literature by expanding the relative paucity of published data on this patient group. It also investigated their physical health, health-related quality of life, recovery-related quality of life, mental wellbeing, experiences of restrictiveness in secure care, cognitive ability and demographics. Despite these important contributions, findings should be seen as exploratory given the relatively low sample size.

In some respects, our findings align with other cross-sectional studies of this group. Other studies also report: lower proportion of women patients than that reflected in the total forensic population ( Coid *et al.*, 2002; women are approximately 18% of the total forensic inpatient population in England and Wales, see Tomlin *et al.*, 2021); a high proportion of serious offences against the person (e.g. murder/manslaughter, assault) ( Di Lorito *et al.*, 2018b); multiple chronic physical health needs alongside complex mental health needs ( Girardi *et al.*, 2018b), and cognitive impairment and high rates of obesity as classified by BMI ( Di Lorito *et al.*, 2019).

On this last point, and considering cardiovascular health more broadly, a recent review of cardiometabolic disease in patients with psychosis of all ages in secure settings reported a weighted pooled prevalence of BMI scores >30 across eight studies of 39.8% (n= 1359); five studies of which reported a weighted pooled prevalence of BMI scores >25 at 72.4% (n= 840) ( Ma *et al.*, 2020). Weighted pooled prevalence scores were also reported for metabolic syndrome: 23.5% (k= 5; n= 1,390); diabetes: 11.3% (k= 12; n= 2,561); dyslipidaemia: 29.2% (k= 8; n= 1,135); hypertension: 25% (k=5; n= 857); cardiovascular disease: 15.6% (k=6; n= 1,047). Further longitudinal research should investigate cardiovascular health across age ranges using the same measures and diagnostic tools, to explore in what ways cardiovascular health might change in secure settings as patients age. The negative relationship between mild cognitive impairment scores and recovery-related quality of life was significant with a moderate effect size, whilst there was a trend towards significance with mental wellbeing. Cognitive ability scores were commensurate to a representative sample of adults living in Ireland with primary level or no education ( Kenny *et al.*, 2013). Using the MoCA threshold of 25/30 or below to indicate possible mild cognitive impairment ( Nasreddine, 2017), we found that 22/37 (65%) of our sample could have mild cognitive impairment. Di Lorito & colleagues (2019) found that 21% of their sample had ‘cognitive impairment’ as measured on the CAMCOG. The mild cognitive impairment scores of our sample are similar to population norms for adults in Ireland who have primary or no education, and lower than adults with secondary or tertiary education (the closest norm values we could find; Kenny *et al*. (2013)) Older forensic patients with poorer cognitive skills will likely need greater support both in hospital and in the community to achieve their recovery goals. A meta-analysis of 49 RCTs comparing interventions to improve global cognition in individuals with mild cognitive impairment found the following intervention types were significantly more effective than control conditions: cognition-based, physical exercise, combined cognition-based and physical exercise, and antioxidants ( Xu *et al.*, 2021). Cognitive Stimulation Therapy (CST) has been suggested in the literature as an example of a cognition-based intervention for this population that can be undertaken via computer or tablet and be facilitated with the support of carers and is not resource intensive ( Natarajan & Mulvana, 2017).

Recovery-related quality of life scores were associated with more positive perceptions of/satisfaction with ‘usual activities’ ^5^. This relationship had a large effect size. This finding underscores the importance of meaningful and accessible activities in the recovery process. Secure inpatient settings have a limited range of activities and community patients can face age-, physical health- or forensic-related barriers to participation ( de Smet *et al.*, 2015). Activities that are available to inpatients have been described as childish or boring or repetitive by older patients in our study ( Walker *et al.*, 2022) and elsewhere ( Di Lorito *et al.*, 2018a; Visser *et al.*, 2021). Patients in the community also describe a paucity of appropriate and engaging activities ( de Smet *et al.*, 2015). Novel, patient-led, and health and age needs-appropriate activities for older patients should be a priority and can be inexpensive to implement compared to complex psychosocial or pharmacological interventions. 

To provide a sense of how our sample compared to other groups, we look to the published literature and population norm values regarding recovery-related quality of life, mental wellbeing, and experiences of restrictiveness. Interestingly, compared to a sample of UK general mental health patients receiving care across different settings (mean= 21.9) our sample (mean= 25.7) has higher mean recovery-related quality of life scores on the ReQoL and lower scores to a representative sample of the UK general population (mean= 28.5) ( Keetharuth *et al.* (2018). We can also see that our sample had similar mental wellbeing scores to the general population as measured by the SWEMWS (mean=23.5) ( Stewart-Brown *et al.*, 2009). When compared to a sample used to develop the FRQ (mean= 35.6), reported similar scores on the measure of patient experiences of restrictiveness in secure care to the sample in Tomlin *et al.* (2019). These comparisons should be investigated in further research using random samples and inferential statistics. Comparing our sample to population norms or published study data, this study found that older forensic patients subjectively rate their mental wellbeing at a level that is not significantly different from the general population ( Stewart-Brown *et al.*, 2009), and their recovery-related quality of life as not significantly different from the general population and better than adults receiving general mental healthcare ( Keetharuth *et al.*, 2018). Although forensic patients have complex comorbid physical and mental health needs, the level of healthcare assessment, monitoring and support they receive is likely to contribute to explaining the lack of significant differences from the general population on these measures. Qualitative studies of patients’ experiences of care suggest that this group likely has better access to healthcare, professional support, social contact, structured activities, regular food, and exercise equipment than the same age group in the community in the general population ( di Lorito *et al.*, 2018a; di Lorito *et al.*, 2017; Visser *et al.*, 2021; Walker *et al.*, 2022). In line with this, mental wellbeing did not correlate with the ‘mobility’, ‘self-care’, and ‘pain/discomfort’ domains of the EQ-5D-5L.

The link between physical health and mental health was not entirely clear though, as the ‘anxiety and depression’ domain of the EQ-5D-5L did correlate with these three domains, and the ReQoL correlated with the ‘mobility’ EQ-5D-5L domain. This could mean that perceptions of ‘depression and anxiety’ are associated with physical health in a way the broader construct of mental wellbeing is not, or that this reflects a response bias given that these domains were all measured on the same questionnaire (the EQ-5D-5L). Nevertheless, given this and the link between mobility and recovery-related quality of life, the associations between physical health and general mental health should be investigated further and services should ensure barriers to mobility are removed.

Age was unrelated to the outcomes measured in this study. This might be because all our patients were 55 years and older (maximum of 70 years), offering little variance for the statistical analysis. One possible explanation for this null finding is that several qualitative investigations have found age to be a subjective construct for many forensic mental health patients; some reject the ‘older’ label and express *feeling* young ( Visser *et al.*, 2021). Interestingly, studies using staff-rated instruments have found that older patients were less likely to have healthcare needs met ( Das *et al.*, 2012); and were less likely to improve over the course of treatment on measures of security needs, self-harm, harm to others, mental health disturbance, personal wellbeing, emotional wellbeing, and socio-economic status ( Girardi *et al.*, 2018).

In relation to medication, the mean number of psychotropic drugs prescribed (2.1 per patient) does not seem excessive given the range and number of diagnoses. The higher total number of drugs (mean = 7.6 per patient) doubtless reflects the burden of physical morbidity, especially diabetes and cardiovascular conditions, in the sample. Of concern is that the mean anticholinergic effect score was 2.4, which suggests that there is scope for review of these medications as they are known to contribute to the future risk of dementia ( Coupland *et al.*, 2019) and are associated with higher risk of mortality and emergency hospitalisation in people with established cognitive impairment ( Bishara *et al.*, 2020).

### Clinical and research implications

To summarise the practical implications of our study, 65% of our sample had possible cognitive impairment according to a validated measure. This suggests that older patients might benefit from interventions to improve cognition or ameliorate cognitive decline, though more evidence is needed to speak to the efficacy of different interventions. Studies suggest improvement might be best achieved through cognition-based interventions, physical exercise and antioxidants ( Xu *et al.*, 2021). Patient recovery-related quality of life and mental wellbeing is likely enhanced by engagement in a range of meaningful and age- or needs-appropriate activities that include work, study, housework, family or leisure activities. Services should continue to address physical healthcare needs, especially relating to cardiovascular health, as patients progress into the community to ensure that physical health concerns do not hinder mental wellbeing and recovery.

The negative correlation of recovery-related quality of life with problems related to depression and anxiety, ability to perform usual activities (work, study, housework, family or leisure) and mobility highlights the importance of seeking to address these issues to enhance quality of life. To reduce levels of obesity and diabetes, more consideration should be given to improving patient physical activity levels, diet, and sleep quality. Acknowledging that many patients will be experiencing cognitive impairment, services should make allowances for this in provision of services, needs assessment, risk assessment, interventions, and treatment, as well as providing relevant staff training.

Further research should address participation in meaningful activities in more detail. Tools that measure aspects of engagement in occupational activities such as the Model of Human Occupation Screening Tool (MOHOST; see: Fan *et al.*, 2016) or the Engagement in Meaningful Activity Survey (EMAS; see: O’ Flynn *et al.*, 2018) should be used. These studies should be longitudinal and compare age groups. This would complement the growing literature assessing levels of met and unmet need in this population ( Girardi *et al.*, 2018). Other research could evaluate CST-based interventions, potentially delivered via iPad app, in this group.

### Limitations

Our study has some limitations. Our sample size can be considered relatively small ( *N*=37), precluding the use of multivariate analysis (e.g. regression) or comparisons of mean differences across multiple groups (e.g. ANOVA). It is possible that given the small sample size, factors not included in this study or not controlled for played a role in shaping patient experiences of for example, quality of life and wellbeing. Having acknowledged this, post-hoc power analyses indicate that most of the analyses included in the study attain the generally accepted power of 0.8 and our sample size is comparable to other studies of this population ( Coid *et al.*, 2002; Das *et al.*, 2011; Das *et al.*, 2012; Di Lorito *et al.*, 2019; Girardi *et al.*, 2018; Lightbody *et al.*, 2010; Tomar *et al.*, 2005), most of which are retrospective using hospital records and did not involve active participant recruitment. The internal consistency of the MoCA was questionable (α=.67), falling just below the generally accepted α=.70 ( Bland & Altman, 1997). Thus, conclusions concerning mild cognitive impairment in this study should be read with some caution. Nonetheless, our findings in this regard are similar to other studies ( Di Lorito *et al.*, 2019). Finally, we did not compare our sample to a representative or whole population of all older patients in England and Wales or a representative or whole population sample of all patients; nor did we obtain a random sample of participants. This places limitations on the extent to which we can conclude our findings are generalisable to patients outside our sample.

## Conclusion

In consultation with a lived experience advisory panel to identify our most important research foci, we investigated correlates of patients’ health-related quality of life, recovery-related quality of life and mental wellbeing. We found that recovery-related quality of life was significantly associated with a measure of mild cognitive impairment and problems engaging in usual activities. Mental wellbeing was trending towards a significant relationship with problems engaging in usual activities ( *p*=0.54). Perceptions of physical health were largely though not entirely uncorrelated to either of these constructs (the exception being recovery-related quality of life and mobility). Age was not correlated with health-related quality of life, recovery-related quality of life or mental wellbeing. There were high levels of possible mild cognitive impairment. Diabetes, vitamin D deficiency, and musculoskeletal and cardiovascular conditions were prevalent. We suggest services co-develop with patients age-appropriate meaningful activities that are mindful of mobility issues and consider implementing cognitive stimulation therapies. This study adds to the growing and much needed literature on older forensic mental health patients and further promotes the importance of studying different marginalised patient groups.

## Data availability

### Underlying data

Open Science Framework: Older adult forensic mental health patients: defining needs, barriers, facilitators and 'what works' to enable better quality of life, health and wellbeing and to reduce risk, https://doi.org/10.17605/OSF.IO/GS37Y. ( Tomlin *et al.,* 2022)

This project contains the following underlying data:

- 2021.11.07 ENHANCE Data presented in Older forensic mental healthcare patients in England- Demographics, physical health, mental wellbeing, cognitive ability and quality of life.sav

### Extended data

Open Science Framework: Older adult forensic mental health patients: defining needs, barriers, facilitators and 'what works' to enable better quality of life, health and wellbeing and to reduce risk, https://doi.org/10.17605/OSF.IO/GS37Y. ( Tomlin *et al.,* 2022)

This project contains the following extended data:

- 2022.01.19 Tomlin *et al.* 2022 Correlations Table.pdf

Data are available under the terms of the Creative Commons Attribution 4.0 International license (CC-BY 4.0).
