# Older forensic mental healthcare patients in England: demographics, physical health, mental wellbeing, cognitive ability and quality of life [version 2; peer review: 2 approved]

**DOI:** 10.3310/nihropenres.13248.1

**Published:** 2022-06-22

**Authors:** Jack Tomlin, Kate Walker, Jen Yates, Tom Dening, Birgit Völlm, Chris Griffiths

**Affiliations:** 1 School of Law and Criminology, University of Greenwich, London, UK; 2 Innovation and Research Department, Northamptonshire Healthcare NHS Foundation Trust, Northampton, UK; 3 Division of Psychiatry and Applied Psychology, School of Medicine, University of Nottingham, Nottingham, UK; 4 Department of Forensic Psychiatry, University Hospital Rostock, Rostock, Germany

**Keywords:** Forensic mental health, older patients, quality of health, mental wellbeing, recovery

## Abstract

**Background:**

Older individuals (e.g., 55 years and over) constitute a growing proportion of the forensic mental health patient population. As a group, they are vulnerable to health outcomes similar to other individuals with serious mental disorders of the same age; however, these concerns can be compounded by complex forensic-related care backgrounds and clinical presentations, lengthy periods of time spent in prison or psychiatric hospitals, substance use histories, and crime perpetration or victimisation. The healthcare needs and strengths of this group are not well understood.

The aim of this study was to identify and describe the demographic, physical health, mental wellbeing, cognitive ability, and quality of life profiles of older forensic patients in community, low, medium, and high security settings in England.

**Methods:**

A cross-sectional quantitative study design was used. N=37 forensic patients aged 55 years and over completed six questionnaires. Data were also collected from patient records.

**Results:**

Most patients were male and were diagnosed with psychosis. The most frequently committed index offence types were violent offences. Patients were prescribed 7.6 medications on average and had average anticholinergic effect on cognition scores of 2.4. Nearly half the sample had diabetes, with an average BMI score of 31.7 (indicating obesity). Possible cognitive impairment was identified in 65% of the sample. Patients’ assessments of their recovery-related quality of life and mental wellbeing were comparable to published UK general population values. Assessments of quality of life were positively correlated with the ability to undertake everyday activities and cognitive performance.

**Conclusions:**

We suggest that forensic services are well-placed to provide holistic mental and physical care to this group but that they should co-develop with patients a greater range of age-appropriate meaningful activities that are mindful of mobility issues and consider implementing more cognition-based and physical health interventions.

## Introduction

Providing support, care and treatment for forensic patients that is responsive to their individual needs and strengths is a core tenet of the recovery approach (Simpson & Penney, 2018). Recognising this requires investigating the individual profiles of patients in forensic mental health services and how patients’ backgrounds, characteristics, experiences and perspectives differ. This approach can be seen in recent efforts to develop services and interventions responsive to the lived experiences, strengths and needs of, amongst others, women patients (de Vogel & Nicholls, 2016), culturally and ethnically diverse patients (Hui, 2017), Deaf patients (Wakeland *et al*., 2019), and older patients (Solares *et al*., 2020). This latter group was the focus of the ENHANCE study^1^, which investigated the demographics, health-related quality of life, recovery rated quality of life, mental wellbeing, cognitive ability, wellbeing and secure hospital restrictiveness rating profiles of forensic mental healthcare patients aged 55 and over.

Forensic mental healthcare patients aged 50 and over constitute about 20% of the UK forensic mental healthcare inpatient population (Di Lorito *et al*., 2019; Di Lorito *et al*., 2018b). This proportion is likely to increase as the population ages (World Health Organization, 2017); indeed, the number of forensic inpatients over 65 in Scotland increased by 50% and those aged 56−65 by 27% between 2013 and 2019 (Scottish Government, 2021). The physical and mental healthcare needs of this group are complex as often comorbidities are prevalent (Natarajan & Mulvana, 2017). Many forensic mental healthcare patients have biographies characterised by placements in psychiatric institutions and prisons, and have experiences of substance abuse and long-term serious mental and physical illnesses for which they may have not received appropriate treatment or management (Centre for Mental Health, 2010; Centre for Mental Health, 2013). This constellation of factors means that many forensic patients experience an ‘accelerated aging’, presenting with a level of health need at, for example, 50 years old that would be equivalent to an average member of the general public at 60 years (Merkt *et al*., 2020).

Compared to younger adult mental healthcare patients, older individuals are more likely to have a higher number of unmet health needs and to experience fewer improvements in their health over time (Das *et al*., 2011; Das *et al*., 2012; Girardi *et al*., 2018). Older patients are more likely to be diagnosed with depression, organic brain syndrome, or delusional disorder (Coid *et al*., 2002). Disabling health issues are more prevalent in older patients; these include cognitive decline, mobility problems and sensory impairment (Di Lorito *et al*., 2019). The number of medications given to older patients has been found to double throughout placement in secure hospitals, highlighting a decline in health (Lightbody *et al*., 2010). Investigating and documenting the disparities between younger and older mental healthcare patients can better equip us to shape service provision, co-develop responsive and appropriate interventions with patients, and address structural disparities in health and wellbeing outcomes (Hui *et al*., 2021).

## Aims and rationale of this study

Despite the growing research and clinical interest in older adult forensic mental health patients there remains a paucity of research data. The current study describes the quality of life, physical health, mental wellbeing, cognitive ability, demographics, and experiences of restrictiveness in secure care of 37 forensic mental healthcare patients, aged 55 and older, from community and low, medium and high security inpatient settings in England.

The research aims were co-authored with the ENHANCE study’s Lived Experience Advisory Panel (LEAP) (a group made up of people with lived experience of mental health issues and forensic mental healthcare service use). These aims were to investigate whether physical health, health-related quality of life, and recovery-related quality of life were correlated with each other in this population, and whether these constructs were associated with: mild cognitive impairment, age, length of stay in secure care, amount of leave (for inpatients), experiences of secure hospital restrictiveness, and treatment setting (i.e. community or low, medium or high security in-patient hospital).

## Methods

### Sampling and recruitment

A stratified cluster sampling frame was planned for community, low and medium secure units, taking into account gender mix and specialisation (e.g. patients with personality disorders or intellectual disabilities). This was disrupted due to COVID-19; however, we were able to recruit participants from a variety of settings across a geographically diverse range of sites. NHS Trusts were recruited through the Clinical Research Network (CRN). No specialist facilities were recruited, sites provided low, medium, and or high secure facilities and community care. Of the 12 community patients recruited, 11 were living independently, with one living in supported accommodation.

Local investigators liaised with members of the study team to identify patients aged over 55 years. These patients were then approached by local investigators to ascertain interest in participation, provide information sheets, and answer any questions about the study. Inclusion criteria for patients was those: aged 55 or over; under the care of forensic mental health services; able to complete self-report questionnaires and semi-structured interviews; who understood written and oral English; and who had capacity to consent. The CRN and PIs at different sites undertook recruitment and initial consenting for participation and as a result it was unknown how patients were approached and how many refused. The number of participants recruited from each trust is depicted in Table 1.

55 years was chosen as the cut-off as this reflects the expedited ageing experienced by forensic patients, suggested by some to be around 10 years (Merkt *et al*., 2020), which aligns with an often-used older age threshold in non-forensic populations used in research of 65 years. The ENHANCE study also included interviews with staff members from these services (reported separately) and patients known to these professionals were invited to take part. All participants were able to provide informed consent to complete questionnaires and to be interviewed. A sample size of N=36 was sought as this was considered sufficient to achieve saturated themes in the qualitative aspects of this project (reported elsewhere) and post-hoc power analyses report the obtained power of the correlations investigated in this study (see ‘Data analysis’).

### Ethical approval

Ethical approval was granted by the NHS Health Research Authority (IRAS: 258016; REC: 19/EM/0350). Funding was provided by the National Institute for Health Research [PB-PG-1217-20028].

### Data collection

Data collection took place between March 2020 and September 2021 across eight National Health Service (NHS) trusts. In total, 38 patients were recruited and completed all study questionnaires. However, it transpired one patient was 53 years old, so their data are excluded from the analysis. Meetings between the researcher responsible for data collection and participants took place in person (*n*=10), via video call (*n*=26), or over the phone (*n*=1). All patients gave informed consent, with written consent taken from those met face-to-face, and verbal recorded consent from those met via video call or phone. Both methods of recording consent were approved by the relevant ethics committee.

Clinical, legal and demographic data (Tomlin *et al*., 2022) were extracted from patient clinical records by principal investigators based at each recruitment site. Legal data included length of stay in the service, nature of the index offence(s) and Mental Health Act 1983 status. Index offences were categorised according to the UK Home Office Offence Classification Index. Where a patient had more than one index offence, we report the most severe as indicated by the Home Office Crime Severity Score. It should be noted that in England and Wales, patients do not need to have committed an index offence to receive treatment in forensic services. They might receive treatment in these services under a civil, non-forensic legal section where they are at risk of harm to themselves or others, which cannot be safely managed in general psychiatric settings.

Clinical data included ICD-10 diagnoses, body mass index (BMI), lists of physical health conditions and medication data (total number of drugs currently prescribed, number of psychotropic drugs currently prescribed, and Anticholinergic Effects on Cognition scores (Bishara *et al*., 2017)). Medications were included if prescribed for regular consumption. As required (*pro re nata* or prn) prescriptions were not counted as it would not be possible to ascertain how much of the drug had actually been administered.

Patients completed six questionnaires:

Q1. The Short Warwick-Edinburgh Mental Wellbeing Scale (SWEMWBS; Tennant *et al*., 2007). This is an overall mental wellbeing measure, consisting of seven self-report questions and Likert scale responses. Higher total ‘metric scores’ indicate better mental wellbeing. Metric scores range from 7 to 35. A score of 18 or less is indicative of probable clinical depression and scores of >18−20 are indicative of possible mild depression.

Q2. The EQ-5D-5L (Devlin *et al*., 2018) is a measure of overall health-related quality of life. It has one self-report question asking for a ‘health today’ score (a higher score indicates better health), and five self-report questions targeting the domains: ‘mobility’, ‘self-care’, ‘usual activities’, ‘pain and discomfort’, and ‘anxiety and depression’ (higher scores on these domains indicate a greater number of problems). Likert scale responses are used. Researchers calculate an ‘index score’, which summarises responses across these five domains. Index scores range from just under 0 to 1, with scores under 0 indicating health states equivalent to or worse than death and 1 suggesting good health (Devlin *et al*., 2018).

Q3. The Recovering Quality of Life measure (ReQoL-10; Keetharuth *et al*., 2018) is an overall recovery-related quality of life questionnaire. This has 10 self-report questions with Likert scale responses. Higher scores indicate more positive quality of life. Scores range from 0 to 40, with scores up to 24 representing the clinical range and scores 25 and greater reflecting the general population.

Q4. The Cambridge Contextual Reading Test − Short Version (Short CCRT; Beardsall, 1998). This is a reading task measure of premorbid IQ, wherein respondents must read sentences aloud that include difficult to pronounce words. Scores are calculated by noting the number of incorrectly pronounced words, such as ‘bouquet’, ‘thyme’ or ‘subtle’. Higher scores indicate better performance. Scores range from 0 to 25.

Q5. The Montreal Cognitive Assessment (MoCA; Nasreddine *et al*., 2005) is a measure of cognitive impairment. It captures respondents’ performance across the domains: attention and concentration, executive functions, memory, language, visuoconstructional skills, conceptual thinking, calculations, and orientation. A total score out of 30 can be calculated, with higher scores indicating better performance. A score <26 indicates possible mild cognitive impairment.

Q6. The Forensic Restrictiveness Questionnaire (FRQ; Tomlin *et al*., 2019). This measures inpatients’ experiences of the restrictiveness of secure inpatient care across 15, self-report Likert scale items. Higher scores indicate greater levels of perceived restrictiveness. Scores range from 15 to 75.

Due to disruptions due to COVID-19, our data collection methods had to be revised during data collection. Two sets of data were collected in person, where the participants completed the four self-report questionnaires (Q1 EQ-5D-5L; Q2 ReQol; Q3 SWEMWBS; Q4 FRQ), and the researcher undertook the two (Q5 MoCA; and Q6 CCRT) other questionnaires with the participants (Q5 and Q6 both require the researcher to actively administer them). The rest of the questionnaires were collected over video-call. For this, the researcher administered Q5 and Q6 to the participants, but Q1−Q4 were filled in by the participants independently to the call. Q1−Q4 were distributed to the participants by principal investigators on each site and were posted/emailed back to the research team once completed.

### Data analysis

Post-hoc power analyses were conducted as the number of completed questionnaires varied (e.g., SWEMWBS *n* =36, and FRQ *n* =27). G*Power (Faul *et al*., 2007) suggested that at *r* = 0.5, *p* = 0.05, two-tailed, n = 36, our analysis yielded a sufficient power of 0.89. G*Power also indicated at *r* = 0.5, *p* = 0.05, two-tailed, n = 27, that analyses including the FRQ yielded a power of 0.78, just under the usually accepted standard of 0.8 (in this study achieved with a sample of *n* =28). This suggests that whilst most correlations conducted in this study are sufficiently powered, our findings involving the FRQ should be seen as exploratory.

Three percent of the questionnaire data were missing, largely due to nine patients not completing the Short CCRT, reporting difficulties with eyesight, reading level or not wanting to complete this. Eight percent of the demographic, legal and clinical data were missing due to recording issues in patient files. Pairwise deletion was used to handle missing data. IBM’s statistical package for the social sciences (SPSS) software v.27 was used. The distribution of questionnaire response data was assessed with the Shapiro-Wilk statistic and most variables were non-normally distributed. To account for this, non-parametric methods were used.

The internal consistency of the questionnaires used in the study was assessed prior to analysis. This suggested all questionnaires were appropriate to use: SWEMWBS, α=.886, *n*=36; EQ-5D-5L, α=.871, *n*=36; ReQoL, α=.859, *n*=36; FRQ, α=.945, *n*=27; and MoCA, α=.660, *n*=34. The alpha value (α) for the MoCA was lower than for the other measures and much of the literature (Nasreddine *et al*., 2005) but other studies (Bernstein *et al*., 2011) have reported values lower than α=.7 (the recommended cut-off for assuming adequate internal consistency; Bland & Altman, 1997). Spearman’s RHO (*ρ*) was used to assess correlations^2^. Effect sizes (*r*) were judges as follows: 0.1, 0.3, and 0.5 as small, medium and large (Cohen, 1988). Statistical significance was set at *p*=<0.05; effect sizes are reported where appropriate. All correlation coefficients reported without a *p* value are significant at *p*=<0.001.

## Results

### Descriptive statistics

Table 2 presents the demographic, clinical and legal profiles of the patient group. Table 3 and Table 4 give an overview of the mental health and physical diagnoses given to patients in the sample. These show that participants were mostly men (92%), of white British ethnicity (81%), with a mean age of 60 years. Median length of stay in current institution was 1404 days (approximately 45 months). The most frequently diagnosed mental disorders were schizophrenia, schizotypal, and delusional disorders (60%), any type of personality disorder (41%), and then mental and behavioural disorders due to psychoactive substance use and mood (affective) disorders (both at 16.2%)^3^. Scores for each questionnaire are presented in Table 5.

### Mental wellbeing

Mental wellbeing (SWEMWBS) was significantly positively associated with recovery-related quality of life (ReQoL) (*ρ*=.773), and ‘health today’ (*ρ*=.486)^4^ as assessed by the EQ-5D-5L, both considered at or above large effect sizes. It was negatively correlated with the depression and anxiety domain (*ρ*= -.348) of the EQ-5D-5L and was trending towards a significant relationship with the ‘usual activities’ domain of the same measure (meaning fewer problems in these domains; *ρ*= -.324, p=.054). A negative correlation was also observed for inpatient perceptions of restrictiveness in care (*ρ*= -.481)), a large effect but the small sample size must be borne in mind here. The association between mental wellbeing and mild cognitive impairment was trending towards significance in a negative direction (*ρ*= -.320, *p*=.065).

### Recovery-related quality of life

Recovery-related quality of life positively correlated with the EQ-5D-5L ‘health today’ domain (*ρ*=.627), an above large effect size, and its overall index value (*ρ*=.362). Recovery-related quality of life was negatively correlated with ‘mobility’ (*ρ*= -.340), ‘usual activities’ (*ρ*= -.551), ‘depression and anxiety’ domains on the EQ-5D-5L (*ρ*= -.408) implying fewer problems on these domains, and (for inpatients) experiences of restrictiveness (*ρ*= -.608), a large effect but the small sample size must be borne in mind here. The association between recovery-related quality of life and mild cognitive impairment was also significant in a negative direction (*ρ*= -.377).

### Health status and perceptions of physical wellbeing

Nearly half (49%) our sample were diagnosed with diabetes, 38% had a disease of the cardiovascular system, and one-fifth (19%) had high cholesterol (e.g. hypercholesterolemia, hyperlipidaemia, raised triglycerides). Around 16% had COPD and 14% had some form of visual impairment. BMI data were available for 30 patients; the mean score for our sample was 31.7, classified as ‘obesity class one’ by the World Health Organisation (WHO). Nine patients met the threshold for ‘pre-obesity’, and 19 patients for obesity classes one, two or three. Only two patients were in the ‘normal weight’ range. On average, patients were prescribed 2.1 psychotropic medications for regular use, with an average anticholinergic effect on cognition score of 2.4, according to the scoring system described in (Bishara *et al*., 2017).

The EQ-5D-5L domains mobility, self-care, usual activities, pain and discomfort, anxiety and depression were all significantly positively correlated with each other. They were all also negatively correlated with the broader EQ-5D-5L indicators of ‘health today’ and the overall ‘index value’ (a measure of overall wellbeing). Patients’ assessment of their health on that specific day was linked with cognitive impairment scores (*ρ*= -.432) whilst their overall health index score on the same questionnaire was not.

### Demographic characteristics and patient outcomes

Age was not significantly linked to any of the outcomes measured. Length of stay in current setting was positively associated with usual activities (*ρ*=.440) and anxiety and depression (*ρ*=.384) indicating a greater number of problems in these domains, and negatively associated with EQ-5D-5L index value suggesting poorer health (*ρ*= -.374). Despite the correlation with the index value, there was no significant relationship with patients’ assessments of their health on that specific day.

Mild cognitive impairment scores were negatively correlated with recovery-related quality of life scores (*ρ*= -.377) and positively with higher restrictiveness ratings (for inpatients, *ρ*=.474). Premorbid IQ was negatively correlated with the EQ-5D-5L ‘health today’ domain (*ρ*= -.432). As our sample was too small to conduct analyses of difference between more than two groups (i.e., ANOVA), we present median recovery-related quality of life, overall wellbeing (EQ-5D-5L index value), mental wellbeing, and experiences of restrictiveness scores across treatment settings and levels of leave in Table 6. Though it is not possible to draw firm conclusions from these findings, at face value there appears to be a trend indicating that scores improve as levels of security decrease from high to low and as levels of leave increase. The exception to this is that community patients appear to have equivalent or poorer outcomes on the SWEMWBS and EQ-5D-5L than inpatients, and ReQoL scores at a level between patients in medium and low security.

## Discussion

This article describes a sample of 37 forensic mental health patients aged 55 and over in forensic community and inpatient mental healthcare services. It makes a novel contribution to the literature by expanding the relative paucity of published data on this patient group. It also investigated their physical health, health-related quality of life, recovery-related quality of life, mental wellbeing, experiences of restrictiveness in secure care, cognitive ability and demographics. Despite these important contributions, findings should be seen as exploratory given the relatively low sample size.

In some respects, our findings align with other cross-sectional studies of this group. Other studies also report: lower proportion of women patients than that reflected in the total forensic population (Coid *et al*., 2002; women are approximately 18% of the total forensic inpatient population in England and Wales, see Tomlin *et al*., 2021); a high proportion of serious offences against the person (e.g. murder/manslaughter, assault) (Di Lorito *et al*., 2018b); multiple chronic physical health needs alongside complex mental health needs (Girardi *et al*., 2018b), and cognitive impairment and high rates of obesity as classified by BMI (Di Lorito *et al*., 2019).

On this last point, and considering cardiovascular health more broadly, a recent review of cardiometabolic disease in patients with psychosis of all ages in secure settings reported a weighted pooled prevalence of BMI scores >30 across eight studies of 39.8% (n= 1359); five studies of which reported a weighted pooled prevalence of BMI scores >25 at 72.4% (n= 840) (Ma *et al*., 2020). Weighted pooled prevalence scores were also reported for metabolic syndrome: 23.5% (k= 5; n= 1,390); diabetes: 11.3% (k= 12; n= 2,561); dyslipidaemia: 29.2% (k= 8; n= 1,135); hypertension: 25% (k=5; n= 857); cardiovascular disease: 15.6% (k=6; n= 1,047). Further longitudinal research should investigate cardiovascular health across age ranges using the same measures and diagnostic tools, to explore in what ways cardiovascular health might change in secure settings as patients age. The negative relationship between mild cognitive impairment scores and recovery-related quality of life was significant with a moderate effect size, whilst there was a trend towards significance with mental wellbeing. Cognitive ability scores were commensurate to a representative sample of adults living in Ireland with primary level or no education (Kenny *et al*., 2013). Using the MoCA threshold of 25/30 or below to indicate possible mild cognitive impairment (Nasreddine, 2017), we found that 22/37 (65%) of our sample could have mild cognitive impairment. Di Lorito & colleagues (2019) found that 21% of their sample had ‘cognitive impairment’ as measured on the CAMCOG. The mild cognitive impairment scores of our sample are similar to population norms for adults in Ireland who have primary or no education, and lower than adults with secondary or tertiary education (the closest norm values we could find; Kenny et al. (2013)) Older forensic patients with poorer cognitive skills will likely need greater support both in hospital and in the community to achieve their recovery goals. A meta-analysis of 49 RCTs comparing interventions to improve global cognition in individuals with mild cognitive impairment found the following intervention types were significantly more effective than control conditions: cognition-based, physical exercise, combined cognition-based and physical exercise, and antioxidants (Xu *et al*., 2021). Cognitive Stimulation Therapy (CST) has been suggested in the literature as an example of a cognition-based intervention for this population that can be undertaken via computer or tablet and be facilitated with the support of carers and is not resource intensive (Natarajan & Mulvana, 2017).

Recovery-related quality of life scores were associated with more positive perceptions of/satisfaction with ‘usual activities’^5^. This relationship had a large effect size. This finding underscores the importance of meaningful and accessible activities in the recovery process. Secure inpatient settings have a limited range of activities and community patients can face age-, physical health- or forensic-related barriers to participation (de Smet *et al*., 2015). Activities that are available to inpatients have been described as childish or boring or repetitive by older patients in our study (Walker *et al*., 2022) and elsewhere (Di Lorito *et al*., 2018a; Visser *et al*., 2021). Patients in the community also describe a paucity of appropriate and engaging activities (de Smet *et al*., 2015). Novel, patient-led, and health and age needs-appropriate activities for older patients should be a priority and can be inexpensive to implement compared to complex psychosocial or pharmacological interventions.

To provide a sense of how our sample compared to other groups, we look to the published literature and population norm values regarding recovery-related quality of life, mental wellbeing, and experiences of restrictiveness. Interestingly, compared to a sample of UK general mental health patients receiving care across different settings (mean= 21.9) our sample (mean= 25.7) has higher mean recovery-related quality of life scores on the ReQoL and lower scores to a representative sample of the UK general population (mean= 28.5) (Keetharuth et al. (2018). We can also see that our sample had similar mental wellbeing scores to the general population as measured by the SWEMWS (mean=23.5) (Stewart-Brown *et al*., 2009). When compared to a sample used to develop the FRQ (mean= 35.6), reported similar scores on the measure of patient experiences of restrictiveness in secure care to the sample in Tomlin et al. (2019). These comparisons should be investigated in further research using random samples and inferential statistics. Comparing our sample to population norms or published study data, this study found that older forensic patients subjectively rate their mental wellbeing at a level that is not significantly different from the general population (Stewart-Brown *et al*., 2009), and their recovery-related quality of life as not significantly different from the general population and better than adults receiving general mental healthcare (Keetharuth *et al*., 2018). Although forensic patients have complex comorbid physical and mental health needs, the level of healthcare assessment, monitoring and support they receive is likely to contribute to explaining the lack of significant differences from the general population on these measures. Qualitative studies of patients’ experiences of care suggest that this group likely has better access to healthcare, professional support, social contact, structured activities, regular food, and exercise equipment than the same age group in the community in the general population (di Lorito *et al*., 2018a; di Lorito *et al*., 2017; Visser *et al*., 2021; Walker *et al*., 2022). In line with this, mental wellbeing did not correlate with the ‘mobility’, ‘self-care’, and ‘pain/discomfort’ domains of the EQ-5D-5L.

The link between physical health and mental health was not entirely clear though, as the ‘anxiety and depression’ domain of the EQ-5D-5L did correlate with these three domains, and the ReQoL correlated with the ‘mobility’ EQ-5D-5L domain. This could mean that perceptions of ‘depression and anxiety’ are associated with physical health in a way the broader construct of mental wellbeing is not, or that this reflects a response bias given that these domains were all measured on the same questionnaire (the EQ-5D-5L). Nevertheless, given this and the link between mobility and recovery-related quality of life, the associations between physical health and general mental health should be investigated further and services should ensure barriers to mobility are removed.

Age was unrelated to the outcomes measured in this study. This might be because all our patients were 55 years and older (maximum of 70 years), offering little variance for the statistical analysis. One possible explanation for this null finding is that several qualitative investigations have found age to be a subjective construct for many forensic mental health patients; some reject the ‘older’ label and express *feeling* young (Visser *et al*., 2021). Interestingly, studies using staff-rated instruments have found that older patients were less likely to have healthcare needs met (Das *et al*., 2012); and were less likely to improve over the course of treatment on measures of security needs, self-harm, harm to others, mental health disturbance, personal wellbeing, emotional wellbeing, and socio-economic status (Girardi *et al*., 2018).

In relation to medication, the mean number of psychotropic drugs prescribed (2.1 per patient) does not seem excessive given the range and number of diagnoses. The higher total number of drugs (mean = 7.6 per patient) doubtless reflects the burden of physical morbidity, especially diabetes and cardiovascular conditions, in the sample. Of concern is that the mean anticholinergic effect score was 2.4, which suggests that there is scope for review of these medications as they are known to contribute to the future risk of dementia (Coupland *et al*., 2019) and are associated with higher risk of mortality and emergency hospitalisation in people with established cognitive impairment (Bishara *et al*., 2020).

### Clinical and research implications

To summarise the practical implications of our study, 65% of our sample had possible cognitive impairment according to a validated measure. This suggests that older patients might benefit from interventions to improve cognition or ameliorate cognitive decline, though more evidence is needed to speak to the efficacy of different interventions. Studies suggest improvement might be best achieved through cognition-based interventions, physical exercise and antioxidants (Xu *et al*., 2021). Patient recovery-related quality of life and mental wellbeing is likely enhanced by engagement in a range of meaningful and age- or needs-appropriate activities that include work, study, housework, family or leisure activities. Services should continue to address physical healthcare needs, especially relating to cardiovascular health, as patients progress into the community to ensure that physical health concerns do not hinder mental wellbeing and recovery.

The negative correlation of recovery-related quality of life with problems related to depression and anxiety, ability to perform usual activities (work, study, housework, family or leisure) and mobility highlights the importance of seeking to address these issues to enhance quality of life. To reduce levels of obesity and diabetes, more consideration should be given to improving patient physical activity levels, diet, and sleep quality. Acknowledging that many patients will be experiencing cognitive impairment, services should make allowances for this in provision of services, needs assessment, risk assessment, interventions, and treatment, as well as providing relevant staff training.

Further research should address participation in meaningful activities in more detail. Tools that measure aspects of engagement in occupational activities such as the Model of Human Occupation Screening Tool (MOHOST; see: Fan *et al*., 2016) or the Engagement in Meaningful Activity Survey (EMAS; see: O’ Flynn *et al*., 2018) should be used. These studies should be longitudinal and compare age groups. This would complement the growing literature assessing levels of met and unmet need in this population (Girardi *et al*., 2018). Other research could evaluate CST-based interventions, potentially delivered via iPad app, in this group.

### Limitations

Our study has some limitations. Our sample size can be considered relatively small (*N*=37), precluding the use of multivariate analysis (e.g. regression) or comparisons of mean differences across multiple groups (e.g. ANOVA). It is possible that given the small sample size, factors not included in this study or not controlled for played a role in shaping patient experiences of for example, quality of life and wellbeing. Having acknowledged this, post-hoc power analyses indicate that most of the analyses included in the study attain the generally accepted power of 0.8 and our sample size is comparable to other studies of this population (Coid *et al*., 2002; Das *et al*., 2011; Das *et al*., 2012; Di Lorito *et al*., 2019; Girardi *et al*., 2018; Lightbody *et al*., 2010; Tomar *et al*., 2005), most of which are retrospective using hospital records and did not involve active participant recruitment. The internal consistency of the MoCA was questionable (α=.67), falling just below the generally accepted α=.70 (Bland & Altman, 1997). Thus, conclusions concerning mild cognitive impairment in this study should be read with some caution. Nonetheless, our findings in this regard are similar to other studies (Di Lorito *et al*., 2019). Finally, we did not compare our sample to a representative or whole population of all older patients in England and Wales or a representative or whole population sample of all patients; nor did we obtain a random sample of participants. This places limitations on the extent to which we can conclude our findings are generalisable to patients outside our sample.

## Conclusion

In consultation with a lived experience advisory panel to identify our most important research foci, we investigated correlates of patients’ health-related quality of life, recovery-related quality of life and mental wellbeing. We found that recovery-related quality of life was significantly associated with a measure of mild cognitive impairment and problems engaging in usual activities. Mental wellbeing was trending towards a significant relationship with problems engaging in usual activities (*p*=0.54). Perceptions of physical health were largely though not entirely uncorrelated to either of these constructs (the exception being recovery-related quality of life and mobility). Age was not correlated with health-related quality of life, recovery-related quality of life or mental wellbeing. There were high levels of possible mild cognitive impairment. Diabetes, vitamin D deficiency, and musculoskeletal and cardiovascular conditions were prevalent. We suggest services co-develop with patients age-appropriate meaningful activities that are mindful of mobility issues and consider implementing cognitive stimulation therapies. This study adds to the growing and much needed literature on older forensic mental health patients and further promotes the importance of studying different marginalised patient groups.

## Figures and Tables

**Table 1:** Number of recruits by participating NHS trusts.

	Total number of recruits	Security levels
Trust 1	4	C = 4
Trust 2	4	MS = 3 LS = 1
Trust 3	4 (3 included, 1 excluded)	MS = 1 LS = 3 (1 excluded)
Trust 4	14	HS = 8 MS = 4 C = 2
Trust 5	4	LS = 2 C = 2
Trust 6	4	LS = 2 C = 2
Trust 7	2	C = 2
Trust 8	2	MS = 1 LS = 1

Notes: C = community; L = low security; MS = medium security; HS = high security

**Table 2:** Demographic, clinical and legal characteristics of the sample.

Characteristic	Frequency / mean / median	% / SD / 25th & 75th
**Age** (*n*=37)	*Mn*= 59.8	*SD*= 3.9
**Sex** (*n*=37)		
- Men	34	92
- Women	3	8
**Ethnicity** (*n*=37)		
- White	30	81
- Black, African, Caribbean, or Black British	6	16
- Mixed or multiple ethnic group	1	3
**Mental Health Act 1983 section** (*n*=37)		
- No legal section (community treatment)	5	14
- s. 3 (civil admission for treatment)	3	8
- s. 37/41 (hospital order and restriction order)	11	30
- s. 37/42 (hospital order and lifted restriction order)	1	3
- s. 41 (treatment in the community and restriction order)	2	5
- s. 41 (5) (notional hospital order)	2	5
- s. 42 (treatment in the community and lifted restriction order)	2	5
- s. 45 (A) (hybrid treatment order)	1	3
- s. 47/49 (prison transfer and restriction order)	9	24
- s. 117 (aftercare following discharge)	1	3
**Index offence** (*n*=37)		
- (Attempted) Murder / Manslaughter	11	30
- Violence against the person	8	21
- Sexual offences	8	21
- Robbery	2	5
- Possession of weapons	1	3
- Threatening to destroy or damage property	1	3
- No offence	6	16
**Setting** (*n*=37)		
- Community	10	27
- Low secure	8	22
- Medium secure	9	24
- High secure	10	27
**Length of stay in days** (*n*=32)	*Mdn*= 1404	25^th^= 469; 75^th^= 3803
**Number of current prescribed medications** (*n*=37)	*Mn*= 7.6	*SD*= 4.4
**Number of current prescribed psychotropic medications** (*n*=37)	*Mn*= 2.1	*SD* = 1.5
**Anticholinergic effect of medications on cognition scores** (*n*=37)	*Mn*= 2.4	*SD* = 2.1
**Body Mass Index** (BMI; *n*=30)	*Mn*= 31.7	*SD* = 4.5
**Possible mild cognitive impairment according to MoCA** (*n*=34)		
- Yes	22	65
- No	12	35

Notes. Percentages of observed values, i-e-excluding missing values. *Mdn,* median; *Mn,* mean; SD, standard deviation; 25^th^ and 75^th^ percentiles.

**Table 3:** Mental health diagnoses in the sample.

Diagnoses ordered by ICD-10 categories	Frequency	%
Organic, including symptomatic, mental disorders	1	2.7
Mental and behavioural disorders due to psychoactive substance use	5	13.5
Schizophrenia, schizotypal and delusional disorders	22	59.5
Mood [affective] disorders	6	16.2
Neurotic, stress-related and somatoform disorders	3	8.1
Personality disorders (Any)	15	40.5
- Dissocial	5	13.5
- Dependent	3	8.1
- Avoidant (anxious)	5	13.5
- Emotionally Unstable	4	10.8
- Paranoid	4	10.8
- Schizoid	2	5.4
- Antisocial	4	10.8
- Borderline	3	8.1
- Mixed Personality Disorder	2	5.4
Disorders of sexual preference	1	2.7
Disorders of psychological development	2	5.4

Notes. Observations greater than 37 and percentages greater than 100 as most patients had multiple diagnoses. *N*=37.

**Table 4:** Physical health burden of the sample.

Physical diagnoses	Frequency	%
Diabetes	18	48.7
Cardiovascular and circulatory system	14	37.8
High cholesterol (e.g. hypercholesterolemia, hyperlipidaemia, raised triglycerides)	7	18.9
Chronic obstructive pulmonary disease (COPD)	6	16.2
Visual impairment	5	13.5
Asthma	4	10.8
Vitamin D deficiency	4	10.8
Diseases of the musculoskeletal system and connective tissue	3	8.1
Hearing loss	1	2.7
Impaired Physical Mobility	1	2.7

Notes. *N*=37.

**Table 5:** Questionnaire scores of the sample.

Questionnaire	Mean	SD
SWEMWBS Metric Score (*n*=36)	23.5	6.3
ReQoL Total Score (*n*=36)	25.7	8.9
EQ-5D-5L Index Value (*n*=36)	0.6	0.4
FRQ Total Score (*n*=27)	32.9	15.7
MoCa Total Score (*n*=34)	23.5	3.8
Short CCRT (*n*=28)	19.3	4.7

**Table 6:** Mental health and wellbeing across treatment settings and levels of leave.

	ReQoL	SWEMWBS Metric	EQ-5D-5L Index	FRQ
Level of Leave				
None	20.5 (12.25)	21.165 (7.59)	.647 (.523)	32.5 (23.5)
Escorted	30 (13)	24.11 (4.48)	.760 (.253)	31 (24)
Unescorted	31 (11.5)	25.065 (15.23)	.884 (.361)	23.5 (42.5)
Setting				
Community	26.5 (18.25)	23.21 (12.43)	.429 (.698)	-
Low	30 (10)	24.62 (11.82)	.836 (.451)	27.5 (36)
Medium	22 (13.5)	22.35 (6.51)	.760 (.166)	30 (11)
High	20 (18)	23.21 (10.28)	.647 (.610)	36 (38.5)

Notes: Median values (interquartile ranges). No FRQ values reported for community patients as the FRQ is a measure of inpatient experience.

## Data Availability

Open Science Framework: Older adult forensic mental health patients: defining needs, barriers, facilitators and ‘what works’ to enable better quality of life, health and wellbeing and to reduce risk, https://doi.org/10.17605/OSF.IO/GS37Y. (Tomlin *et al*., 2022) This project contains the following underlying data: - 2021.11.07 ENHANCE Data presented in Older forensic mental healthcare patients in England-Demographics, physical health, mental wellbeing, cognitive ability and quality of life.sav Open Science Framework: Older adult forensic mental health patients: defining needs, barriers, facilitators and ‘what works’ to enable better quality of life, health and wellbeing and to reduce risk, https://doi.org/10.17605/OSF.IO/GS37Y. (Tomlin *et al*., 2022) This project contains the following extended data: - 2022.01.19 Tomlin *et al.* 2022 Correlations Table.pdf Data are available under the terms of the Creative Commons Attribution 4.0 International license (CC-BY 4.0).

## Amendments from Version 1

We have revised the manuscript following the helpful feedback of two reviewers. These revisions to a large extent involved explaining in more detail our process of recruitment and data collection and analysis. We no longer use inferential statistics to compare our sample to other published samples and instead make a comparison at face-value, signposting future research in this area.

**Any further responses from the reviewers can be found at the end of the article**

## Plain language summary

Forensic mental health services provide care for people who have committed a crime or are at risk of harm to themselves or others. This care takes many forms, including mental health support, physical health care, and assistance to re-enter the community after spending time in secure mental health hospitals. Due to changes in the general population, there is a growing number of patients over the age of 55 in care. Despite this, we do not know enough about the healthcare needs of this older patient group and how these needs differ from younger patients. This study aimed to find out more about this patient group.

In the current study, thirty-seven forensic patients aged 55 years and older were recruited from low, medium and high security hospitals and community care. These patients completed six questionnaires. Patients were asked about their physical health, mental wellbeing, cognitive ability, and quality of life. Information about medical diagnoses and socio-demographic backgrounds were collected from hospital records.

The study found that these patients had complex health needs. Most patients were diagnosed with psychosis (e.g., schizophrenia), were men, and had committed a violent criminal offence. On average patients were prescribed 7.6 medications, many of which carry significant side effects. Nearly half of the patients had diabetes, and most patients were obese or overweight. Quality of life scores were lower for patients who also reported having problems undertaking everyday activities and patients with cognitive impairment. Patients subjectively rated their mental wellbeing and quality of life similarly to the general population’s ratings as reported in other studies. We suggest that services should work with patients to develop a greater number of age-appropriate activities and interventions aimed at improving cognitive and physical health.

## Open Peer Review

**Current Peer Review Status**: ✔ ✔

**Version 2**

https://doi.org/10.3310/nihropenres.14408.r28694

**© 2022 Goethals K.** This is an open access peer review report distributed under the terms of the Creative Commons Attribution License, which permits unrestricted use, distribution, and reproduction in any medium, provided the original work is properly cited.

✔ **Kris Goethals**
  Department of Psychiatry, Collaborative Antwerp Psychiatric Research Institute, Antwerp, Belgium
I am happy with the answers of the authors and the amendments they have made. In my opinion this manuscript is ready for indexing now.
***Competing Interests:*** No competing interests were disclosed.
**I confirm that I have read this submission and believe that I have an appropriate level of expertise to confirm that it is of an acceptable scientific standard.**

https://doi.org/10.3310/nihropenres.14408.r28693

**© 2022 MacInnes D.** This is an open access peer review report distributed under the terms of the Creative Commons Attribution License, which permits unrestricted use, distribution, and reproduction in any medium, provided the original work is properly cited.

✔ **Douglas MacInnes**^iD^
  Faculty of Medicine, Health and Social Care, Canterbury Christ Church University, Canterbury, UK
The authors have considered my comments made in relation to version 1 and addressed these in a thoughtful and constructive manner. As noted in my previous comments, I think this is an important topic area. The revisions made result in a greater focus on the data collected from the 37 participants. This has resulted in the paper reporting interesting and valuable findings which help to develop our understanding of the issues surrounding caring for this group of service users.
***Competing Interests:*** No competing interests were disclosed.
***Reviewer Expertise:*** Mental Health and particularly forensic psychiatry, stigma, social networks, co-production and working with families and carers
**I confirm that I have read this submission and believe that I have an appropriate level of expertise to confirm that it is of an acceptable scientific standard**.

**Version 1**

https://doi.org/10.3310/nihropenres.14364.r28480

**© 2022 Goethals K.** This is an open access peer review report distributed under the terms of the Creative Commons Attribution License, which permits unrestricted use, distribution, and reproduction in any medium, provided the original work is properly cited.

**？** **Kris Goethals**
  Department of Psychiatry, Collaborative Antwerp Psychiatric Research Institute, Antwerp, Belgium
This paper shows interesting research that is well written and structured. The patient number is low, all of them are forensic psychiatric patients.
  ⚬ Can you explain in the text why the patient number is so low, since you recruited them from a range of forensic mental health settings in England? I think this needs some clarification.
  ⚬ Seven of them had no offences. Can you explain this lack of offences? All of them are forensic psychiatric patients?
  ⚬ In my opinion there is an overload of figures in the manuscript (figures 1 and 2). Maybe you could select some of them and explain more in the body of the paper (without figures).
**Is the work clearly and accurately presented and does it cite the current literature?**
  Yes
**Is the study design appropriate and is the work technically sound?**
  Yes
**Are sufficient details of methods and analysis provided to allow replication by others?**
  Partly
**If applicable, is the statistical analysis and its interpretation appropriate?**
  I cannot comment. A qualified statistician is required.
**Are all the source data underlying the results available to ensure full reproducibility?**
  Yes
**Are the conclusions drawn adequately supported by the results?**
  Yes
***Competing Interests:*** No competing interests were disclosed.
**I confirm that I have read this submission and believe that I have an appropriate level of expertise to confirm that it is of an acceptable scientific standard, however I have significant reservations, as outlined above.**

Author Response 15 Jun 2022 **Jack Tomlin,** University of Greenwich, London, UK This paper shows interesting research that is well written and structured. The patient number is low, all of them are forensic psychiatric patients. Can you explain in the text why the patient number is so low, since you recruited them from a range of forensic mental health settings in England? I think this needs some clarification.
**We would like to thank the reviewer for your time and comments.**

**The sample is small as the number of older forensic patients (>55) is generally low; it is quite resources intensive to involve new sites or 'NHS Trusts' within a single study, so we had to cap the number of participating sites due to this; patients were recruited only where giving consent, so we could not use hospital records to ascertain information of all eligible patients; and finally, the quantitative data were collected in the same meeting as a qualitative interview was undertaken, with the quantitative questionnaires seen as a complementary to the qualitative interviews. Only collecting quantitative data might have increased the willingness of patients to participate and reduced the resource intensiveness of the study, allowing more time to recruit participants. To make this clearer in the manuscript, we have added to the section 'Sampling and recruitment":**

**"The study aimed to recruit N=36 participants so as to reach saturation of themes in the concurrent qualitative analysis and this was deemed sufficient for basic statistical analysis of association between variables in the quantitative analysis presented in this paper; post-hoc power calculations are presented below."**
Seven of them had no offences. Can you explain this lack of offences? All of them are forensic psychiatric patients?
**This is an important point to clarify, thank you. We have added the following to the 'Data collection' section:**

**"It should be noted that in England and Wales, patients do not need to have committed an index offence to receive treatment in forensic services. They might receive treatment in these services under a civil, non-forensic legal section where they are at risk of harm to themselves or others, which cannot be safely managed in general psychiatric settings."**
In my opinion there is an overload of figures in the manuscript (figures 1 and 2). Maybe you could select some of them and explain more in the body of the paper (without figures).
**This is helpful observation; a similar point was made by Reviewer 1. Accordingly, we have removed these figures and replaced them with Table 4. Also following comments from Reviewer 1, we have added some more information on the information include in Table 4. We have sought to explain in more detail these findings in the 'Results' section:**

**"As our sample was too small to conduct analyses of difference between more than two groups (i.e., ANOVA), we present median recovery-related quality of life, overall wellbeing (EQ-5D-5L index value), mental wellbeing, and experiences of restrictiveness scores across treatment settings and levels of leave in Table 4. Though it is not possible to draw firm conclusions from these findings, at face value there appears to be a trend indicating that scores become preferable as levels of security decrease from high to low and as levels of leave increase. The exception to this is that community patients appear to have equivalent or poorer outcomes on the SWEMWBS and EQ-5D-5L than inpatients, and ReQoL scores at a level between patients in medium and low security."**
***Competing Interests***: None

https://doi.org/10.3310/nihropenres.14364.r28473

**© 2022 MacInnes D.** This is an open access peer review report distributed under the terms of the Creative Commons Attribution License, which permits unrestricted use, distribution, and reproduction in any medium, provided the original work is properly cited.

✖ Douglas MacInnes^iD^
  Faculty of Medicine, Health and Social Care, Canterbury Christ Church University, Canterbury, UK
The paper looks at an important topic; older people in forensic mental health settings, with the number of older adults increasing in number in secure settings. It is primarily an exploratory paper reporting descriptive demographic, clinical and forensic data concerning the 37 patients in the study. The paper then examines the strengths of relationships between some of these variables with a set of 6 outcome measures. There is then an attempt to broaden out the paper with inferential analysis used to compare the data from this study with data from populations norms and published samples which I think is not warranted (and will discuss this further below). It would be better as a more focused paper acknowledging it is a small-scale descriptive study with an examination of associations between variables. I would suggest the paper should focus on the data obtained from these 37 participants and the findings. This would lead to some tentative conclusions being able to be made but not any broad generalisations about the wider older patient population in forensic settings.
I have made a number of comments that I think would be helpful for the authors to address. I hope they are useful.
**Abstract**
  It notes the research design is a mixed methods approach. Although the authors note that qualitative work was undertaken as part of the larger study, this paper does not include any of this and so either the research design noted needs to be changed or the other element(s) of the mixed methods approach needs to be included.
**Defining an older patient in forensic settings**
  In the introduction, there is some mention of previous studies where 50+ mentioned as the cut-off age for examining older adults in these settings. However, none seem to have 55+ as a cut-off. It would be helpful to detail why 55+ was chosen as the age to decide who should be viewed as an older patient as well as acknowledging alternative definitions and the implications of using 55 as a cut-off.
**Recruitment**
  Further information is needed about the recruitment process. How was the recruitment managed? 8 separate trusts were included and it would be helpful to be given some information about these 8 sites. How many older patients were recruited from these different sites? Were any specialist facilities i.e. (older people, women, physical needs)? What were the community facilities? Were there differences in recruitment rates between the sites? How many were approached? How many refused to take part? What was the inclusion/exclusion criteria for participants? How many were excluded through this? Were there any differences noted in the between those recruited and those older patients in these sites. How did the demographic and clinical and forensic characteristics compare to the 1500 or so older patients in secure mental health facilities in England and Wales? These questions also relate to the representativeness of the study sample and how generalisable the study findings are to this wider group of older forensic patients. Some discussions about this would be beneficial.
**Interview data**
  The use of interviews to gather qualitative data was mentioned on a number of occasions as well as noting this was not part of the study. It is probably best to mention this once (that interviews were conducted but qualitative data not part of the study) and then remove any other comments about interviews.
**Data Collection**
  The six self-report measures were noted on pg4. It would be useful to ensure that the scoring range of these measures are detailed. It is also noted that a score of <26 on the MoCA indicates possible cognitive impairment. Could you state whether any of the other measures have cut-off points? Are there any other cut-off points for determining level of wellness and, if possible, record these in your results? It would also be useful to describe how the data were collected particularly as different approaches were used (i.e. how many in in person, via phone etc) and document whether there were any differences in the approach used (i.e. did any participant require help, was anyone with them when they undertook the assessments, were the measures always completed in a set or random order).
**Sample size calculation**
  On pg5 it is noted that Power Calculations were undertaken using G*Power. I presume this was through calculating the sample size needed for a Pearsons R correlation. It would be helpful if you could give further details about the procedures used when determining that a minimum sample size of 36 was required (I got a proposed minimum sample size of 46 when I did this calculation). In addition, three of the measures (FRQ, MoCA and short CCRT) reported smaller numbers of participants' answering the measure than the proposed minimum sample size of 36. Could you give a rationale for including these measures in the analysis? Will this have reduced the likelihood of important associations being found?
**Aims**
  It was a little confusing to note the specific aims and objectives of the study. It is stated on pg3 the aim was "to better understand the profiles of this patient group and investigate factors associated with quality of life, recovery and well-being". However, a revised set of aims is detailed on pg4. To help the reader, one coherent set of aims and objectives should be noted.
**Results**
  Correlations - It would be helpful to give some indication of the criteria you used to determine the strength of relationship between the variables (I presume you used Cohen's conventions). There are a number of interesting findings. A number of these reported are moderate associations. Would it be worth focusing more on the stronger relationships reported? This would be supported by the likelihood that a small number of outlier scores could influence the rho scores due to the small sample size. In addition, the estimated effect size of 0.5 noted as a parameter in the power calculation suggested that the predicted relationship effect would be large.
  A small point; some Spearmans rho scores are reported with associated probability values whilst others were. It would be better to adopt one consistent approach.
**Figures**
  I am unsure about the figures 1 and 2 and what they bring to the paper. The small numbers in each category make it extremely unlikely that any significant differences would be recorded and the boxplots, visually, don't show any meaningful differences in scores. As an example, in Fig1 (pg9), the differences in scores between those with no leave allowed and those on unescorted leave appears to be 10 points. This is a significant clinical difference even if it is not statistically significant as is a ReQoL score <24 recorded by the "no leave allowed" group. I think a table reporting the median and inter-quartile range would give much clearer information. Part of the discussion about the use of also looked at the potential use of ANOVA. It was noted that the data set would not allow ANOVA to be used but on a wider level the use of statistical analysis to examine differences in in scores is at odds with the analysis undertaken in the paper which focused on examining at the strength of the relationships between variables. Focusing on ether the relationship or difference is preferable.
**Comparison with populations norms and published samples**
  I think it is very difficult to use the findings in this study and compare them with populations norms and published samples apart from noting the scores of this study sample and scores recorded by by other samples. Even then, any comparisons should be tentative as there are many extraneous variables that may have impacted on this study's findings. As an example, I think it would be impossible to make any relevant comparisons between the cognitive abilities of this group of participants and the educational attainment of students in Ireland without additional data about the educational attainment achieved by the 37 participants. I would also strongly suggest that carrying out inferential analysis is wrong and misleading and should be removed from the findings as it can induce misleading findings.
**Discussion**
  It may be helpful to focus on the main findings reported by the 37 participants and, in particular, where there were large strength of associations recorded.
  The issues with regards to limited sample size should included in the discussions (i.e. relationship of age to outcomes reported). The samples are being reported as a homogenous group but the limited sample size means that the scores may be majorly influenced by other factors (i.e. quality of life scores can be strongly influenced by the service being received, the clinical environment and therapeutic engagement).
  Pg 10 - When discussing cardiovascular health, it is mentioned that direct comparisons are not possible due to different methods of diagnosis and recording practice. If this is the case, any comparative discussions should be removed.
  Pg 12 - It is noted that that a men number of 2.1 psychotropic medications does not seem excessive and also that "of concern is the mean anticholinergic effect of 2.4. Some supportive reference for viewing these scores as such would be helpful.
  Pg 12 - It was proposed that a larger sample with patients aged over 18 may have led to different findings. I wasn't sure about what point was being made by this sentence. Some clarification would be helpful.
  Pg 12 - It was reported that some patients may have felt younger and rated a number of outcomes in line with this. If this is the case, how valid are the measures used?
**Implications**
  On pg12, it is stated that our data suggest that older patients could benefit from interventions to improve cognition or ameliorate cognitive decline. This needs to be clearly detailed from the data as, at present, this doesn't seem to be the case - the data only states there is cognitive decline in these participants and it is in the discussion where ways of dealing with this are suggested.
**Limitations**
  These need to be clearly noted. There are issues reading sample size and it is questionable whether the study was sufficiently powered for some of the correlational analysis. The representativeness of the sample and how generalisability of the findings should also be considered
**Conclusions**
  It is suggested on pg13 services co-develop with patients' meaningful activities. I agree with this statement but if it is to be a concluding point, it should be explicitly mentioned in the discussion (or elsewhere in the paper).
**Is the work clearly and accurately presented and does it cite the current literature?**
  Yes
**Is the study design appropriate and is the work technically sound?**
  Partly
**Are sufficient details of methods and analysis provided to allow replication by others?**
  Partly
**If applicable, is the statistical analysis and its interpretation appropriate?**
  No
**Are all the source data underlying the results available to ensure full reproducibility?**
  Yes
**Are the conclusions drawn adequately supported by the results?**
  Partly
***Competing Interests:*** No competing interests were disclosed.
***Reviewer Expertise:*** Mental Health and particularly forensic psychiatry, stigma, social networks, co-production and working with families and carers
**I confirm that I have read this submission and believe that I have an appropriate level of expertise to state that I do not consider it to be of an acceptable scientific standard, for reasons outlined above.**

*Author Response 15 Jun 2022*
**Jack Tomlin,** University of Greenwich, London, UK The paper looks at an important topic; older people in forensic mental health settings, with the number of older adults increasing in number in secure settings. It is primarily an exploratory paper reporting descriptive demographic, clinical and forensic data concerning the 37 patients in the study. The paper then examines the strengths of relationships between some of these variables with a set of 6 outcome measures. There is then an attempt to broaden out the paper with inferential analysis used to compare the data from this study with data from populations norms and published samples which I think is not warranted (and will discuss this further below). It would be better as a more focused paper acknowledging it is a small-scale descriptive study with an examination of associations between variables. I would suggest the paper should focus on the data obtained from these 37 participants and the findings. This would lead to some tentative conclusions being able to be made but not any broad generalisations about the wider older patient population in forensic settings.
**We thank the reviewer for the time they took to review this manuscript and for their helpful comments. We hope we have addressed the reviewer's feedback and have revised the manuscript to a satisfactory standard.**
I have made a number of comments that I think would be helpful for the authors to address. I hope they are useful. Abstract It notes the research design is a mixed methods approach. Although the authors note that qualitative work was undertaken as part of the larger study, this paper does not include any of this and so either the research design noted needs to be changed or the other element(s) of the mixed methods approach needs to be included.
**Thank you, this is noted. This now reads: 'A cross-sectional quantitative study design was used.'**
Defining an older patient in forensic settings In the introduction, there is some mention of previous studies where 50+ mentioned as the cut-off age for examining older adults in these settings. However, none seem to have 55+ as a cut-off. It would be helpful to detail why 55+ was chosen as the age to decide who should be viewed as an older patient as well as acknowledging alternative definitions and the implications of using 55 as a cut-off.
**We agree that this needs clarification. We have added the following text: '55 years was chosen as this reflects the expedited ageing experienced by forensic patients, suggested by some to be around 10 years (Merkt *et al.,* 2020), which aligns with an often-used older age threshold in non-forensic populations used in research of 65 years.'**
Recruitment Further information is needed about the recruitment process. How was the recruitment managed? 8 separate trusts were included and it would be helpful to be given some information about these 8 sites. How many older patients were recruited from these different sites? Were any specialist facilities i.e. (older people, women, physical needs)? What were the community facilities? Were there differences in recruitment rates between the sites? How many were approached? How many refused to take part? What was the inclusion/exclusion criteria for participants? How many were excluded through this? Were there any differences noted in the between those recruited and those older patients in these sites. How did the demographic and clinical and forensic characteristics compare to the 1500 or so older patients in secure mental health facilities in England and Wales? These questions also relate to the representativeness of the study sample and how generalisable the study findings are to this wider group of older forensic patients. Some discussions about this would be beneficial.
**Thank you for highlighting these issues. We agree that more information is needed. We have renamed the section 'Participants', to 'Sampling and recruitment', to which we have added:**

**"A stratified cluster sampling frame was planned for community, low and medium secure units, taking into account gender mix and specialisation (e.g. patients with personality disorders or intellectual disabilities). This was disrupted due to COVID-19; however, we were able to recruit participants from a variety of settings across a geographically diverse range of sites. NHS Trusts were recruited through the Clinical Research Network. No specialist facilities were recruited, sites provided low, medium, and or high secure facilities and community care. Of the 12 community patients recruited, 11 were living independently, with one living in supported accommodation.**

**Local investigators liaised with members of the study team to identify patients aged over 55 years. These patients were then approached by local investigators to ascertain interest in participation, provide information sheets, and answer any questions about the study. Inclusion criteria for patients was those: aged 55 or over; under the care of forensic mental health services; able to complete self-report questionnaires and semi-structured interviews; who understood written and oral English; and who had capacity to consent. The CRN and PIs at different sites undertook recruitment and initial consenting for participation and as a result it was unknown how patients were approached and how many refused. The number of participants recruited from each trust is depicted in Table 1."**
Interview data The use of interviews to gather qualitative data was mentioned on a number of occasions as well as noting this was not part of the study. It is probably best to mention this once (that interviews were conducted but qualitative data not part of the study) and then remove any other comments about interviews.
**This is a good point. We have kept only two mentions of this and removed all others.**
Data Collection The six self-report measures were noted on pg4. It would be useful to ensure that the scoring range of these measures are detailed. It is also noted that a score of <26 on the MoCA indicates possible cognitive impairment. Could you state whether any of the other measures have cut-off points? Are there any other cut-off points for determining level of wellness and, if possible, record these in your results? It would also be useful to describe how the data were collected particularly as different approaches were used (i.e. how many in in person, via phone etc) and document whether there were any differences in the approach used (i.e. did any participant require help, was anyone with them when they undertook the assessments, were the measures always completed in a set or random order).
**Thank you for these helpful comments. We have added descriptions for the score ranges and cut-offs for all questionnaires where these are available. Please see additional sentences added to the descriptions of the questionnaires in the 'Data collection' section.**

**We describe how many different approaches were used to collect data in the section titled 'Data Collection' in paragraph one. E.g., "Meetings between the researcher responsible for data collection and participants took place in person ( n=10), via video call ( n=26), or over the phone ( n=1)."**

**We have added more information on the data collection process, e.g., how questionnaires were completed by participants:**

**"Due to disruptions due to COVID-19, our data collection methods had to be revised during data collection. Two sets of data were collected in person, where the participants completed the four self-report questionnaires (Q1 EQ-5D-5L; Q2 ReQol; Q3 SWEMWBS; Q4 FRQ), and the researcher undertook the two (Q5 MoCA; and Q6 CCRT) other questionnaires with the participants (Q5 and Q6 both require the researcher to actively administer them). The rest of the questionnaires were collected over video-call. For this, the researcher administered Q5 and Q6 to the participants, but Q1-Q4 were filled in by the participants independently to the call. Q1-Q4 were distributed to the participants by principal investigators on each site and were posted/emailed back to the research team once completed."**
Sample size calculation On pg5 it is noted that Power Calculations were undertaken using G*Power. I presume this was through calculating the sample size needed for a Pearsons R correlation. It would be helpful if you could give further details about the procedures used when determining that a minimum sample size of 36 was required (I got a proposed minimum sample size of 46 when I did this calculation). In addition, three of the measures (FRQ, MoCA and short CCRT) reported smaller numbers of participants' answering the measure than the proposed minimum sample size of 36. Could you give a rationale for including these measures in the analysis? Will this have reduced the likelihood of important associations being found?
**This is a great point, and something we have overlooked. As we have different sample sizes as you point out, we conducted post-hoc analyses to investigate the extent to which our findings were sufficiently powered. We have sought to remind readers that our findings should be observed with some degree of caution given the small sample size (e.g., paragraph one of discussion and limitations). We have added the following to the manuscript:**

"Post-hoc power analyses were conducted as the number of completed questionnaires varied (e.g., SWEMWBS n =36, and FRQ n =27). G*Power ( Faul et al., 2007) suggested that at r = 0.5, p = 0.05, two-tailed, n = 36, our analysis yielded a sufficient power of 0.89. G*Power also indicated at r = 0.5, p = 0.05, two-tailed, n = 27, that analyses including the FRQ yielded a power of 0.78, just under the usually accepted standard of 0.8 (in this study achieved with a sample of n =28). This suggests that whilst most correlations conducted in this study are sufficiently powered, our findings involving the FRQ should be seen as exploratory."
Aims It was a little confusing to note the specific aims and objectives of the study. It is stated on pg3 the aim was "to better understand the profiles of this patient group and investigate factors associated with quality of life, recovery and well-being". However, a revised set of aims is detailed on pg4. To help the reader, one coherent set of aims and objectives should be noted. **Thank you for highlighting this. We have removed the following sentence to provide further clarity: 'The aims of this study were to better understand the profiles of this patient group and investigate factors associated with quality of life, recovery, and wellbeing.'**. Results Correlations - It would be helpful to give some indication of the criteria you used to determine the strength of relationship between the variables (I presume you used Cohen's conventions). There are a number of interesting findings. A number of these reported are moderate associations. Would it be worth focusing more on the stronger relationships reported? This would be supported by the likelihood that a small number of outlier scores could influence the rho scores due to the small sample size. In addition, the estimated effect size of 0.5 noted as a parameter in the power calculation suggested that the predicted relationship effect would be large. **Good point - we have now added the effect sizes (Cohen's r) to our 'Data Analysis' section. Indeed, many of the significant correlations were between .3 and .7 so we have chosen a large effect size of .5 for the power calculation. We have signposted readers to large and moderate effect sizes throughout the findings and discussion where appropriate. We think this help make the important findings of the study clearer**. A small point; some Spearmans rho scores are reported with associated probability values whilst others were. It would be better to adopt one consistent approach.
**Thank you for highlighting this. We only report p values of Spearman RHO analyses where these were not significant or were trending towards significance. All other Spearman RHO coefficients reported without a p value are significant at P=<0.001. To clarify, we added the following to the 'Data analysis' section: 'All correlation coefficients reported without a p value are significant at p=<0.001.'**
Figures I am unsure about the figures 1 and 2 and what they bring to the paper. The small numbers in each category make it extremely unlikely that any significant differences would be recorded and the boxplots, visually, don't show any meaningful differences in scores. As an example, in Fig1 (pg9), the differences in scores between those with no leave allowed and those on unescorted leave appears to be 10 points. This is a significant clinical difference even if it is not statistically significant as is a ReQoL score <24 recorded by the "no leave allowed" group. I think a table reporting the median and inter-quartile range would give much clearer information. Part of the discussion about the use of also looked at the potential use of ANOVA. It was noted that the data set would not allow ANOVA to be used but on a wider level the use of statistical analysis to examine differences in in scores is at odds with the analysis undertaken in the paper which focused on examining at the strength of the relationships between variables. Focusing on ether the relationship or difference is preferable.
**Thank you for these comments. We have now revised this section of the findings. Instead of discussing the potential findings of an ANOVA in a future study, we now include a table of median and IQR values and the following text:**

**"As our sample was too small to conduct analyses of difference between more than two groups (i.e., ANOVA), we present median recovery-related quality of life, overall wellbeing (EQ-5D-5L index value), mental wellbeing, and experiences of restrictiveness scores across treatment settings and levels of leave in Table 4. Though it is not possible to draw firm conclusions from these findings, at face value there appears to be a trend indicating that scores become preferable as levels of security decrease from high to low and as levels of leave increase. The exception to this is that community patients appear to have equivalent or poorer outcomes on the SWEMWBS and EQ-5D-5L than inpatients, and ReQoL scores at a level between patients in medium and low security."**
Comparison with populations norms and published samples I think it is very difficult to use the findings in this study and compare them with populations norms and published samples apart from noting the scores of this study sample and scores recorded by by other samples. Even then, any comparisons should be tentative as there are many extraneous variables that may have impacted on this study's findings. As an example, I think it would be impossible to make any relevant comparisons between the cognitive abilities of this group of participants and the educational attainment of students in Ireland without additional data about the educational attainment achieved by the 37 participants. I would also strongly suggest that carrying out inferential analysis is wrong and misleading and should be removed from the findings as it can induce misleading findings.
**We note this feedback and have removed the inferential statistics. We now present a face-value comparison in the discussion and have removed the statistical comparison as an aim of the study. This is evident throughout the manuscript, but please note the largest changes to the 'Discussion' section.**

**"To provide some sense of how our sample compared to other groups, we look to the published literature and population norm values regarding recovery-related quality of life, mental wellbeing, and experiences of restrictiveness. Interestingly, compared to a sample of UK general mental health patients receiving care across different settings (mean= 21.9) our sample (mean= 25.7) has higher mean recovery-related quality of life scores on the ReQoL and lower scores to a representative sample of the UK general population (mean= 28.5) ( Keetharuth et al. (2018). We can also see that our sample had similar mental wellbeing scores to the general population as measured by the SWEMWS (mean=23.5) (Stewart-Brown et al., 2009). When compared to a sample used to develop the FRQ (mean= 35.6), reported similar scores on the measure of patient experiences of restrictiveness in secure care to the sample in Tomlin et al. (2019). These comparisons should be investigated in further research using random samples and inferential statistics."**
Discussion It may be helpful to focus on the main findings reported by the 37 participants and, in particular, where there were large strength of associations recorded.
**We have included mention of effect sizes in the Discussion section, so as to acknowledge the larger associations. We have also scaled back our discussion to keep this closer to the findings of the paper; we have revised heavily the paragraph looking at published population norms or other study data.**
The issues with regards to limited sample size should included in the discussions (i.e. relationship of age to outcomes reported). The samples are being reported as a homogenous group but the limited sample size means that the scores may be majorly influenced by other factors (i.e. quality of life scores can be strongly influenced by the service being received, the clinical environment and therapeutic engagement).
**We have revised this, so that our limitations section now includes the following amended text:**

**"It is possible that given the small sample size, factors not included in this study or not controlled for played a role in shaping patient experiences of for example, quality of life and wellbeing. Having acknowledged this, post-hoc power analyses indicate that most of the analyses included in the study attain the generally accepted power of 0.8 and our sample size is comparable to other studies of this population (Coid et al., 2002; Das et al., 2011; Das et al., 2012; Di Lorito et al., 2019; Girardi et al., 2018; Lightbody et al., 2010; Tomar et al., 2005), most of which are retrospective using hospital records and did not involve active participant recruitment."**
Pg 10 - When discussing cardiovascular health, it is mentioned that direct comparisons are not possible due to different methods of diagnosis and recording practice. If this is the case, any comparative discussions should be removed.
**We have deleted this and replaced this with:**

**"Further longitudinal research should investigate cardiovascular health across age ranges using the same measures and diagnostic tools, to explore in what ways cardiovascular health might change in secure settings as patients age."**
Pg 12 - It is noted that that a men number of 2.1 psychotropic medications does not seem excessive and also that "of concern is the mean anticholinergic effect of 2.4. Some supportive reference for viewing these scores as such would be helpful. **We hope we have clarified this. We provide information on links between anticholinergic effect on cognition with mental wellbeing later in the referenced paragraph. We state that we use the scoring system described by (Bishara et al., 2017) in the sections: 'Health status and perceptions of physical wellbeing' and 'Data collection'**. Pg 12 - It was proposed that a larger sample with patients aged over 18 may have led to different findings. I wasn't sure about what point was being made by this sentence. Some clarification would be helpful. + Pg 12 - It was reported that some patients may have felt younger and rated a number of outcomes in line with this. If this is the case, how valid are the measures used?
**In response to the two points above - we agree that this is confusingly worded. We have sought to simplify this by deleting the following, and simplifying our main point in this paragraph:**

**"As our outcomes were largely subjectively measured it is possible some respondents felt younger or older than others the same age and rated their mental wellbeing, physical health and recovery-related quality of life in line with this. A larger sample with patients aged 18 and older might have led to different findings."**
Implications On pg12, it is stated that our data suggest that older patients could benefit from interventions to improve cognition or ameliorate cognitive decline. This needs to be clearly detailed from the data as, at present, this doesn't seem to be the case - the data only states there is cognitive decline in these participants and it is in the discussion where ways of dealing with this are suggested.
**We agree implications should be couched in sufficient evidence. We have reworded this section to reflect our findings and signpost the need for more research:**

**"To summarise the practical implications of our study, 65% of our sample had possible cognitive impairment according to a validated measure. This suggests that older patients might benefit from interventions to improve cognition or ameliorate cognitive decline, though more evidence is needed to speak to the efficacy of different interventions. Studies suggest improvement might be best achieved through cognition-based interventions, physical exercise and antioxidants (Xu et al., 2021)."**
Limitations These need to be clearly noted. There are issues reading sample size and it is questionable whether the study was sufficiently powered for some of the correlational analysis. The representativeness of the sample and how generalisability of the findings should also be considered
**This is an important point. We have acknowledged this by including the following in the 'Limitations' section:**

**"Finally, we did not compare our sample to a representative or whole population of all older patients in England and Wales or a representative or whole population sample of all patients; nor did we obtain a random sample of participants. This places limitations on the extent to which we can conclude our findings are generalisable to patients outside our sample."**
Conclusions It is suggested on pg13 services co-develop with patients' meaningful activities. I agree with this statement but if it is to be a concluding point, it should be explicitly mentioned in the discussion (or elsewhere in the paper).
**We are glad that the Reviewer agrees that this an important point. As we do not think we can introduce the topic of co-production in detail in this manuscript, we have made a small reference to this in the introduction, as we would like to keep this in:**

**"Investigating and documenting the disparities between younger and older mental healthcare patients can better equip us to shape service provision, co-develop responsive and appropriate interventions with patients, and address structural disparities in health and wellbeing outcomes (Hui et al., 2021)."**
***Competing Interests:*** None

## Footnotes

1 Full name: ‘Older adult forensic mental health patients: defining needs, barriers, facilitators and ‘what works’ to enable better quality of life, health and wellbeing and to reduce risk’

2 Due to potential confounding effects of premorbid verbal IQ on patients’ performance on the study questionnaires, we correlated premorbid verbal IQ with each questionnaire. Where a significant relationship was found, non-parametric correlations and partial correlations controlling for premorbid verbal IQ were undertaken and reported.

3 Percentages greater than 100 as most patients have more than one diagnosis.

4 This relationship remained significant after conducting a partial correlation controlling for the effects of premorbid verbal IQ because our measure of this, the Short CCRT, correlated significantly with the ‘health today’ domain of the EQ-5D-5L.

5 Defined in the EQ-5D-5L as: work, study, housework, family or leisure activities. Mental wellbeing was trending towards a significant relationship with ‘usual activities’ also (p=.054).
