# Supplementary material for: Older forensic mental healthcare patients in England: demographics, physical health, mental wellbeing, cognitive ability and quality of life
Source: NIHR Open Res. 2022 Jun 22;2:9. Originally published 2022 Feb 3. [Version 2] doi: 10.3310/nihropenres.13248.2 (PMC7613860; doi:10.3310/nihropenres.13248.2)
Supplement: Peer review history

# Reviewer report: Reviewer response for version 2

**DOI:** 10.3310/nihropenres.14408.r28694

**Published:** 2022-08-11

**Authors:** Kris Goethals

**Affiliations:** 1 Department of Psychiatry, Collaborative Antwerp Psychiatric Research Institute, Antwerp, Belgium

I am happy with the answers of the authors and the amendments they have made. In my opinion this manuscript is ready for indexing now.

Is the work clearly and accurately presented and does it cite the current literature?

Yes

If applicable, is the statistical analysis and its interpretation appropriate?

I cannot comment. A qualified statistician is required.

Are all the source data underlying the results available to ensure full reproducibility?

Yes

Is the study design appropriate and is the work technically sound?

Yes

Are the conclusions drawn adequately supported by the results?

Yes

Are sufficient details of methods and analysis provided to allow replication by others?

Partly

Reviewer Expertise:

NA

I confirm that I have read this submission and believe that I have an appropriate level of expertise to confirm that it is of an acceptable scientific standard.

# Reviewer report: Reviewer response for version 2

**DOI:** 10.3310/nihropenres.14408.r28693

**Published:** 2022-07-19

**Authors:** Douglas MacInnes

**Affiliations:** 1 Faculty of Medicine, Health and Social Care, Canterbury Christ Church University, Canterbury, UK

The authors have considered my comments made in relation to version 1 and addressed these in a thoughtful and constructive manner. As noted in my previous comments, I think this is an important topic area. The revisions made result in a greater focus on the data collected from the 37 participants. This has resulted in the paper reporting interesting and valuable findings which help to develop our understanding of the issues surrounding caring for this group of service users.

Is the work clearly and accurately presented and does it cite the current literature?

Yes

If applicable, is the statistical analysis and its interpretation appropriate?

No

Are all the source data underlying the results available to ensure full reproducibility?

Yes

Is the study design appropriate and is the work technically sound?

Partly

Are the conclusions drawn adequately supported by the results?

Partly

Are sufficient details of methods and analysis provided to allow replication by others?

Partly

Reviewer Expertise:

Mental Health and particularly forensic psychiatry, stigma, social networks, co-production and working with families and carers

I confirm that I have read this submission and believe that I have an appropriate level of expertise to confirm that it is of an acceptable scientific standard.

# Reviewer report: Reviewer response for version 1

**DOI:** 10.3310/nihropenres.14364.r28480

**Published:** 2022-04-08

**Authors:** Kris Goethals

**Affiliations:** 1 Department of Psychiatry, Collaborative Antwerp Psychiatric Research Institute, Antwerp, Belgium

This paper shows interesting research that is well written and structured. The patient number is low, all of them are forensic psychiatric patients.

- Can you explain in the text why the patient number is so low, since you recruited them from a range of forensic mental health settings in England? I think this needs some clarification.
- Seven of them had no offences. Can you explain this lack of offences? All of them are forensic psychiatric patients?
- In my opinion there is an overload of figures in the manuscript (figures 1 and 2). Maybe you could select some of them and explain more in the body of the paper (without figures).

Is the work clearly and accurately presented and does it cite the current literature?

Yes

If applicable, is the statistical analysis and its interpretation appropriate?

I cannot comment. A qualified statistician is required.

Are all the source data underlying the results available to ensure full reproducibility?

Yes

Is the study design appropriate and is the work technically sound?

Yes

Are the conclusions drawn adequately supported by the results?

Yes

Are sufficient details of methods and analysis provided to allow replication by others?

Partly

Reviewer Expertise:

NA

I confirm that I have read this submission and believe that I have an appropriate level of expertise to confirm that it is of an acceptable scientific standard, however I have significant reservations, as outlined above.

# Response

**Published:** 2022-06-15

**Authors:** Jack Tomlin

**Affiliations:** University of Greenwich, UK

This paper shows interesting research that is well written and structured. The patient number is low, all of them are forensic psychiatric patients. 

 Can you explain in the text why the patient number is so low, since you recruited them from a range of forensic mental health settings in England? I think this needs some clarification. 

**We would like to thank the reviewer for your time and comments.**

****

**The sample is small as the number of older forensic patients (>55) is generally low; it is quite resources intensive to involve new sites or ‘NHS Trusts’ within a single study, so we had to cap the number of participating sites due to this; patients were recruited only where giving consent, so we could not use hospital records to ascertain information of all eligible patients; and finally, the quantitative data were collected in the same meeting as a qualitative interview was undertaken, with the quantitative questionnaires seen as a complementary to the qualitative interviews. Only collecting quantitative data might have increased the willingness of patients to participate and reduced the resource intensiveness of the study, allowing more time to recruit participants. To make this clearer in the manuscript, we have added to the section ‘Sampling and recruitment”:**

**“The study aimed to recruit N=36 participants so as to reach saturation of themes in the concurrent qualitative analysis and this was deemed sufficient for basic statistical analysis of association between variables in the quantitative analysis presented in this paper; post-hoc power calculations are presented below.”**

 Seven of them had no offences. Can you explain this lack of offences? All of them are forensic psychiatric patients? 

**This is an important point to clarify, thank you. We have added the following to the ‘Data collection’ section:**

**“It should be noted that in England and Wales, patients do not need to have committed an index offence to receive treatment in forensic services. They might receive treatment in these services under a civil, non-forensic legal section where they are at risk of harm to themselves or others, which cannot be safely managed in general psychiatric settings.”**

   

 In my opinion there is an overload of figures in the manuscript (figures 1 and 2). Maybe you could select some of them and explain more in the body of the paper (without figures). 

****

**This is helpful observation; a similar point was made by Reviewer 1. Accordingly, we have removed these figures and replaced them with Table 4. Also following comments from Reviewer 1, we have added some more information on the information include in Table 4. We have sought to explain in more detail these findings in the ‘Results’ section:**

**“As our sample was too small to conduct analyses of difference between more than two groups (i.e., ANOVA), we present median recovery-related quality of life, overall wellbeing (EQ-5D-5L index value), mental wellbeing, and experiences of restrictiveness scores across treatment settings and levels of leave in Table 4. Though it is not possible to draw firm conclusions from these findings, at face value there appears to be a trend indicating that scores become preferable as levels of security decrease from high to low and as levels of leave increase. The exception to this is that community patients appear to have equivalent or poorer outcomes on the SWEMWBS and EQ-5D-5L than inpatients, and ReQoL scores at a level between patients in medium and low security.”**

# Reviewer report: Reviewer response for version 1

**DOI:** 10.3310/nihropenres.14364.r28473

**Published:** 2022-03-10

**Authors:** Douglas MacInnes

**Affiliations:** 1 Faculty of Medicine, Health and Social Care, Canterbury Christ Church University, Canterbury, UK

The paper looks at an important topic; older people in forensic mental health settings, with the number of older adults increasing in number in secure settings. It is primarily an exploratory paper reporting descriptive demographic, clinical and forensic data concerning the 37 patients in the study. The paper then examines the strengths of relationships between some of these variables with a set of 6 outcome measures. There is then an attempt to broaden out the paper with inferential analysis used to compare the data from this study with data from populations norms and published samples which I think is not warranted (and will discuss this further below). It would be better as a more focused paper acknowledging it is a small-scale descriptive study with an examination of associations between variables. I would suggest the paper should focus on the data obtained from these 37 participants and the findings. This would lead to some tentative conclusions being able to be made but not any broad generalisations about the wider older patient population in forensic settings.

 I have made a number of comments that I think would be helpful for the authors to address. I hope they are useful.

**Abstract**

 It notes the research design is a mixed methods approach. Although the authors note that qualitative work was undertaken as part of the larger study, this paper does not include any of this and so either the research design noted needs to be changed or the other element(s) of the mixed methods approach needs to be included.

**Defining an older patient in forensic settings**

 In the introduction, there is some mention of previous studies where 50+ mentioned as the cut-off age for examining older adults in these settings. However, none seem to have 55+ as a cut-off. It would be helpful to detail why 55+ was chosen as the age to decide who should be viewed as an older patient as well as acknowledging alternative definitions and the implications of using 55 as a cut-off.

**Recruitment**

 Further information is needed about the recruitment process. How was the recruitment managed? 8 separate trusts were included and it would be helpful to be given some information about these 8 sites. How many older patients were recruited from these different sites? Were any specialist facilities i.e. (older people, women, physical needs)? What were the community facilities? Were there differences in recruitment rates between the sites? How many were approached? How many refused to take part? What was the inclusion/exclusion criteria for participants? How many were excluded through this? Were there any differences noted in the between those recruited and those older patients in these sites. How did the demographic and clinical and forensic characteristics compare to the 1500 or so older patients in secure mental health facilities in England and Wales? These questions also relate to the representativeness of the study sample and how generalisable the study findings are to this wider group of older forensic patients. Some discussions about this would be beneficial.

**Interview data**

 The use of interviews to gather qualitative data was mentioned on a number of occasions as well as noting this was not part of the study. It is probably best to mention this once (that interviews were conducted but qualitative data not part of the study) and then remove any other comments about interviews.

**Data Collection**

 The six self-report measures were noted on pg4. It would be useful to ensure that the scoring range of these measures are detailed. It is also noted that a score of <26 on the MoCA indicates possible cognitive impairment. Could you state whether any of the other measures have cut-off points? Are there any other cut-off points for determining level of wellness and, if possible, record these in your results? It would also be useful to describe how the data were collected particularly as different approaches were used (i.e. how many in in person, via phone etc) and document whether there were any differences in the approach used (i.e. did any participant require help, was anyone with them when they undertook the assessments, were the measures always completed in a set or random order).

**Sample size calculation**

 On pg5 it is noted that Power Calculations were undertaken using G*Power. I presume this was through calculating the sample size needed for a Pearsons R correlation. It would be helpful if you could give further details about the procedures used when determining that a minimum sample size of 36 was required (I got a proposed minimum sample size of 46 when I did this calculation). In addition, three of the measures (FRQ, MoCA and short CCRT) reported smaller numbers of participants’ answering the measure than the proposed minimum sample size of 36. Could you give a rationale for including these measures in the analysis? Will this have reduced the likelihood of important associations being found?

**Aims**

 It was a little confusing to note the specific aims and objectives of the study. It is stated on pg3 the aim was “to better understand the profiles of this patient group and investigate factors associated with quality of life, recovery and well-being”. However, a revised set of aims is detailed on pg4. To help the reader, one coherent set of aims and objectives should be noted.

**Results**

 Correlations – It would be helpful to give some indication of the criteria you used to determine the strength of relationship between the variables (I presume you used Cohen’s conventions). There are a number of interesting findings. A number of these reported are moderate associations. Would it be worth focusing more on the stronger relationships reported? This would be supported by the likelihood that a small number of outlier scores could influence the rho scores due to the small sample size. In addition, the estimated effect size of 0.5 noted as a parameter in the power calculation suggested that the predicted relationship effect would be large.

 A small point; some Spearmans rho scores are reported with associated probability values whilst others were. It would be better to adopt one consistent approach. 

**Figures**

 I am unsure about the figures 1 and 2 and what they bring to the paper. The small numbers in each category make it extremely unlikely that any significant differences would be recorded and the boxplots, visually, don’t show any meaningful differences in scores. As an example, in Fig1 (pg9), the differences in scores between those with no leave allowed and those on unescorted leave appears to be 10 points. This is a significant clinical difference even if it is not statistically significant as is a ReQoL score <24 recorded by the “no leave allowed” group. I think a table reporting the median and inter-quartile range would give much clearer information. Part of the discussion about the use of also looked at the potential use of ANOVA. It was noted that the data set would not allow ANOVA to be used but on a wider level the use of statistical analysis to examine differences in in scores is at odds with the analysis undertaken in the paper which focused on examining at the strength of the relationships between variables. Focusing on ether the relationship or difference is preferable.

**Comparison with populations norms and published samples**

 I think it is very difficult to use the findings in this study and compare them with populations norms and published samples apart from noting the scores of this study sample and scores recorded by by other samples. Even then, any comparisons should be tentative as there are many extraneous variables that may have impacted on this study’s findings. As an example, I think it would be impossible to make any relevant comparisons between the cognitive abilities of this group of participants and the educational attainment of students in Ireland without additional data about the educational attainment achieved by the 37 participants. I would also strongly suggest that carrying out inferential analysis is wrong and misleading and should be removed from the findings as it can induce misleading findings.

**Discussion**

 It may be helpful to focus on the main findings reported by the 37 participants and, in particular, where there were large strength of associations recorded.

 The issues with regards to limited sample size should included in the discussions (i.e. relationship of age to outcomes reported). The samples are being reported as a homogenous group but the limited sample size means that the scores may be majorly influenced by other factors (i.e. quality of life scores can be strongly influenced by the service being received, the clinical environment and therapeutic engagement).

 Pg 10 - When discussing cardiovascular health, it is mentioned that direct comparisons are not possible due to different methods of diagnosis and recording practice. If this is the case, any comparative discussions should be removed.

 Pg 12 - It is noted that that a men number of 2.1 psychotropic medications does not seem excessive and also that “of concern is the mean anticholinergic effect of 2.4. Some supportive reference for viewing these scores as such would be helpful. 

 Pg 12 - It was proposed that a larger sample with patients aged over 18 may have led to different findings. I wasn’t sure about what point was being made by this sentence. Some clarification would be helpful. 

 Pg 12 - It was reported that some patients may have felt younger and rated a number of outcomes in line with this. If this is the case, how valid are the measures used?

**Implications**

 On pg12, it is stated that our data suggest that older patients could benefit from interventions to improve cognition or ameliorate cognitive decline. This needs to be clearly detailed from the data as, at present, this doesn’t seem to be the case – the data only states there is cognitive decline in these participants and it is in the discussion where ways of dealing with this are suggested.

**Limitations**

 These need to be clearly noted. There are issues reading sample size and it is questionable whether the study was sufficiently powered for some of the correlational analysis. The representativeness of the sample and how generalisability of the findings should also be considered

**Conclusions**

 It is suggested on pg13 services co-develop with patients’ meaningful activities. I agree with this statement but if it is to be a concluding point, it should be explicitly mentioned in the discussion (or elsewhere in the paper).

Is the work clearly and accurately presented and does it cite the current literature?

Yes

If applicable, is the statistical analysis and its interpretation appropriate?

No

Are all the source data underlying the results available to ensure full reproducibility?

Yes

Is the study design appropriate and is the work technically sound?

Partly

Are the conclusions drawn adequately supported by the results?

Partly

Are sufficient details of methods and analysis provided to allow replication by others?

Partly

Reviewer Expertise:

Mental Health and particularly forensic psychiatry, stigma, social networks, co-production and working with families and carers

I confirm that I have read this submission and believe that I have an appropriate level of expertise to state that I do not consider it to be of an acceptable scientific standard, for reasons outlined above.

# Response

**Published:** 2022-06-15

**Authors:** Jack Tomlin

**Affiliations:** University of Greenwich, UK

The paper looks at an important topic; older people in forensic mental health settings, with the number of older adults increasing in number in secure settings. It is primarily an exploratory paper reporting descriptive demographic, clinical and forensic data concerning the 37 patients in the study. The paper then examines the strengths of relationships between some of these variables with a set of 6 outcome measures. There is then an attempt to broaden out the paper with inferential analysis used to compare the data from this study with data from populations norms and published samples which I think is not warranted (and will discuss this further below). It would be better as a more focused paper acknowledging it is a small-scale descriptive study with an examination of associations between variables. I would suggest the paper should focus on the data obtained from these 37 participants and the findings. This would lead to some tentative conclusions being able to be made but not any broad generalisations about the wider older patient population in forensic settings. 

**We thank the reviewer for the time they took to review this manuscript and for their helpful comments. We hope we have addressed the reviewer’s feedback and have revised the manuscript to a satisfactory standard.**

****

 I have made a number of comments that I think would be helpful for the authors to address. I hope they are useful. 

 Abstract 

 It notes the research design is a mixed methods approach. Although the authors note that qualitative work was undertaken as part of the larger study, this paper does not include any of this and so either the research design noted needs to be changed or the other element(s) of the mixed methods approach needs to be included. 

**Thank you, this is noted. This now reads: ‘A cross-sectional quantitative study design was used.’**

 Defining an older patient in forensic settings 

 In the introduction, there is some mention of previous studies where 50+ mentioned as the cut-off age for examining older adults in these settings. However, none seem to have 55+ as a cut-off. It would be helpful to detail why 55+ was chosen as the age to decide who should be viewed as an older patient as well as acknowledging alternative definitions and the implications of using 55 as a cut-off. 

**We agree that this needs clarification. We have added the following text: ‘55 years was chosen as this reflects the expedited ageing experienced by forensic patients, suggested by some to be around 10 years (Merkt *et al*., 2020), which aligns with an often-used older age threshold in non-forensic populations used in research of 65 years.’**

 Recruitment 

 Further information is needed about the recruitment process. How was the recruitment managed? 8 separate trusts were included and it would be helpful to be given some information about these 8 sites. How many older patients were recruited from these different sites? Were any specialist facilities i.e. (older people, women, physical needs)? What were the community facilities? Were there differences in recruitment rates between the sites? How many were approached? How many refused to take part? What was the inclusion/exclusion criteria for participants? How many were excluded through this? Were there any differences noted in the between those recruited and those older patients in these sites. How did the demographic and clinical and forensic characteristics compare to the 1500 or so older patients in secure mental health facilities in England and Wales? These questions also relate to the representativeness of the study sample and how generalisable the study findings are to this wider group of older forensic patients. Some discussions about this would be beneficial. 

**Thank you for highlighting these issues. We agree that more information is needed. We have renamed the section ‘Participants’, to ‘Sampling and recruitment’, to which we have added:**

**“A stratified cluster sampling frame was planned for community, low and medium secure units, taking into account gender mix and specialisation (e.g. patients with personality disorders or intellectual disabilities). This was disrupted due to COVID-19; however, we were able to recruit participants from a variety of settings across a geographically diverse range of sites. NHS Trusts were recruited through the Clinical Research Network. No specialist facilities were recruited, sites provided low, medium, and or high secure facilities and community care. Of the 12 community patients recruited, 11 were living independently, with one living in supported accommodation.**

**Local investigators liaised with members of the study team to identify patients aged over 55 years. These patients were then approached by local investigators to ascertain interest in participation, provide information sheets, and answer any questions about the study. Inclusion criteria for patients was those: aged 55 or over; under the care of forensic mental health services; able to complete self-report questionnaires and semi-structured interviews; who understood written and oral English; and who had capacity to consent. The CRN and PIs at different sites undertook recruitment and initial consenting for participation and as a result it was unknown how patients were approached and how many refused. The number of participants recruited from each trust is depicted in Table 1.”**

 Interview data 

 The use of interviews to gather qualitative data was mentioned on a number of occasions as well as noting this was not part of the study. It is probably best to mention this once (that interviews were conducted but qualitative data not part of the study) and then remove any other comments about interviews. 

**This is a good point. We have kept only two mentions of this and removed all others.**

 Data Collection 

 The six self-report measures were noted on pg4. It would be useful to ensure that the scoring range of these measures are detailed. It is also noted that a score of <26 on the MoCA indicates possible cognitive impairment. Could you state whether any of the other measures have cut-off points? Are there any other cut-off points for determining level of wellness and, if possible, record these in your results? It would also be useful to describe how the data were collected particularly as different approaches were used (i.e. how many in in person, via phone etc) and document whether there were any differences in the approach used (i.e. did any participant require help, was anyone with them when they undertook the assessments, were the measures always completed in a set or random order). 

**Thank you for these helpful comments. We have added descriptions for the score ranges and cut-offs for all questionnaires where these are available. Please see additional sentences added to the descriptions of the questionnaires in the ‘Data collection’ section.**

**We describe how many different approaches were used to collect data in the section titled ‘Data Collection’ in paragraph one. E.g., “Meetings between the researcher responsible for data collection and participants took place in person ( n=10), via video call ( n=26), or over the phone ( n=1).”**

**We have added more information on the data collection process, e.g., how questionnaires were completed by participants:**

**“Due to disruptions due to COVID-19, our data collection methods had to be revised during data collection. Two sets of data were collected in person, where the participants completed the four self-report questionnaires (Q1 EQ-5D-5L; Q2 ReQol; Q3 SWEMWBS;  Q4 FRQ), and the researcher undertook the two (Q5 MoCA; and Q6 CCRT) other questionnaires with the participants (Q5 and Q6 both require the researcher to actively administer them). The rest of the questionnaires were collected over video-call. For this, the researcher administered Q5 and Q6 to the participants, but Q1-Q4 were filled in by the participants independently to the call. Q1-Q4 were distributed to the participants by principal investigators on each site and were posted/emailed back to the research team once completed.”**

 Sample size calculation 

 On pg5 it is noted that Power Calculations were undertaken using G*Power. I presume this was through calculating the sample size needed for a Pearsons R correlation. It would be helpful if you could give further details about the procedures used when determining that a minimum sample size of 36 was required (I got a proposed minimum sample size of 46 when I did this calculation). In addition, three of the measures (FRQ, MoCA and short CCRT) reported smaller numbers of participants’ answering the measure than the proposed minimum sample size of 36. Could you give a rationale for including these measures in the analysis? Will this have reduced the likelihood of important associations being found? 

**This is a great point, and something we have overlooked. As we have different sample sizes as you point out, we conducted post-hoc analyses to investigate the extent to which our findings were sufficiently powered. We have sought to remind readers that our findings should be observed with some degree of caution given the small sample size (e.g., paragraph one of discussion and limitations). We have added the following to the manuscript:**

“Post-hoc power analyses were conducted as the number of completed questionnaires varied (e.g., SWEMWBS n =36, and FRQ n =27). G*Power ( Faul et al., 2007) suggested that at r = 0.5, p = 0.05, two-tailed, n = 36, our analysis yielded a sufficient power of 0.89. G*Power also indicated at r = 0.5, p = 0.05, two-tailed, n = 27, that analyses including the FRQ yielded a power of 0.78, just under the usually accepted standard of 0.8 (in this study achieved with a sample of n =28). This suggests that whilst most correlations conducted in this study are sufficiently powered, our findings involving the FRQ should be seen as exploratory.”

 Aims 

 It was a little confusing to note the specific aims and objectives of the study. It is stated on pg3 the aim was “to better understand the profiles of this patient group and investigate factors associated with quality of life, recovery and well-being”. However, a revised set of aims is detailed on pg4. To help the reader, one coherent set of aims and objectives should be noted. 

**Thank you for highlighting this. We have removed the following sentence to provide further clarity: ‘The aims of this study were to better understand the profiles of this patient group and investigate factors associated with quality of life, recovery, and wellbeing.’.**

 Results 

 Correlations – It would be helpful to give some indication of the criteria you used to determine the strength of relationship between the variables (I presume you used Cohen’s conventions). There are a number of interesting findings. A number of these reported are moderate associations. Would it be worth focusing more on the stronger relationships reported? This would be supported by the likelihood that a small number of outlier scores could influence the rho scores due to the small sample size. In addition, the estimated effect size of 0.5 noted as a parameter in the power calculation suggested that the predicted relationship effect would be large. 

**Good point – we have now added the effect sizes (Cohen’s r) to our ‘Data Analysis’ section. Indeed, many of the significant correlations were between .3 and .7 so we have chosen a large effect size of .5 for the power calculation. We have signposted readers to large and moderate effect sizes throughout the findings and discussion where appropriate. We think this help make the important findings of the study clearer.**

 A small point; some Spearmans rho scores are reported with associated probability values whilst others were. It would be better to adopt one consistent approach.  

**Thank you for highlighting this. We only report p values of Spearman RHO analyses where these were not significant or were trending towards significance. All other Spearman RHO coefficients reported without a p value are significant at P=<0.001. To clarify, we added the following to the ‘Data analysis’ section: ‘All correlation coefficients reported without a p value are significant at p=<0.001.’**

 Figures 

 I am unsure about the figures 1 and 2 and what they bring to the paper. The small numbers in each category make it extremely unlikely that any significant differences would be recorded and the boxplots, visually, don’t show any meaningful differences in scores. As an example, in Fig1 (pg9), the differences in scores between those with no leave allowed and those on unescorted leave appears to be 10 points. This is a significant clinical difference even if it is not statistically significant as is a ReQoL score <24 recorded by the “no leave allowed” group. I think a table reporting the median and inter-quartile range would give much clearer information. Part of the discussion about the use of also looked at the potential use of ANOVA. It was noted that the data set would not allow ANOVA to be used but on a wider level the use of statistical analysis to examine differences in in scores is at odds with the analysis undertaken in the paper which focused on examining at the strength of the relationships between variables. Focusing on ether the relationship or difference is preferable. 

**Thank you for these comments. We have now revised this section of the findings. Instead of discussing the potential findings of an ANOVA in a future study, we now include a table of median and IQR values and the following text:**

**“As our sample was too small to conduct analyses of difference between more than two groups (i.e., ANOVA), we present median recovery-related quality of life, overall wellbeing (EQ-5D-5L index value), mental wellbeing, and experiences of restrictiveness scores across treatment settings and levels of leave in Table 4. Though it is not possible to draw firm conclusions from these findings, at face value there appears to be a trend indicating that scores become preferable as levels of security decrease from high to low and as levels of leave increase. The exception to this is that community patients appear to have equivalent or poorer outcomes on the SWEMWBS and EQ-5D-5L than inpatients, and ReQoL scores at a level between patients in medium and low security.”**

 Comparison with populations norms and published samples 

 I think it is very difficult to use the findings in this study and compare them with populations norms and published samples apart from noting the scores of this study sample and scores recorded by by other samples. Even then, any comparisons should be tentative as there are many extraneous variables that may have impacted on this study’s findings. As an example, I think it would be impossible to make any relevant comparisons between the cognitive abilities of this group of participants and the educational attainment of students in Ireland without additional data about the educational attainment achieved by the 37 participants. I would also strongly suggest that carrying out inferential analysis is wrong and misleading and should be removed from the findings as it can induce misleading findings. 

**We note this feedback and have removed the inferential statistics. We now present a face-value comparison in the discussion and have removed the statistical comparison as an aim of the study. This is evident throughout the manuscript, but please note the largest changes to the ‘Discussion’ section.**

**“To provide some sense of how our sample compared to other groups, we look to the published literature and population norm values regarding recovery-related quality of life, mental wellbeing, and experiences of restrictiveness. Interestingly, compared to a sample of UK general mental health patients receiving care across different settings (mean= 21.9) our sample (mean= 25.7) has higher mean recovery-related quality of life scores on the ReQoL and lower scores to a representative sample of the UK general population (mean= 28.5) ( Keetharuth et al. (2018). We can also see that our sample had similar mental wellbeing scores to the general population as measured by the SWEMWS (mean=23.5) ( Stewart-Brown et al., 2009). When compared to a sample used to develop the FRQ (mean= 35.6), reported similar scores on the measure of patient experiences of restrictiveness in secure care to the sample in Tomlin et al. (2019). These comparisons should be investigated in further research using random samples and inferential statistics.”**

 Discussion 

 It may be helpful to focus on the main findings reported by the 37 participants and, in particular, where there were large strength of associations recorded. 

**We have included mention of effect sizes in the Discussion section, so as to acknowledge the larger associations. We have also scaled back our discussion to keep this closer to the findings of the paper; we have revised heavily the paragraph looking at published population norms or other study data.**

 The issues with regards to limited sample size should included in the discussions (i.e. relationship of age to outcomes reported). The samples are being reported as a homogenous group but the limited sample size means that the scores may be majorly influenced by other factors (i.e. quality of life scores can be strongly influenced by the service being received, the clinical environment and therapeutic engagement). 

**We have revised this, so that our limitations section now includes the following amended text:**

**“It is possible that given the small sample size, factors not included in this study or not controlled for played a role in shaping patient experiences of for example, quality of life and wellbeing. Having acknowledged this, post-hoc power analyses indicate that most of the analyses included in the study attain the generally accepted power of 0.8 and our sample size is comparable to other studies of this population ( Coid et al., 2002; Das et al., 2011; Das et al., 2012; Di Lorito et al., 2019; Girardi et al., 2018; Lightbody et al., 2010; Tomar et al., 2005), most of which are retrospective using hospital records and did not involve active participant recruitment.”**

 Pg 10 - When discussing cardiovascular health, it is mentioned that direct comparisons are not possible due to different methods of diagnosis and recording practice. If this is the case, any comparative discussions should be removed. 

**We have deleted this and replaced this with:**

**“Further longitudinal research should investigate cardiovascular health across age ranges using the same measures and diagnostic tools, to explore in what ways cardiovascular health might change in secure settings as patients age.”**

 Pg 12 - It is noted that that a men number of 2.1 psychotropic medications does not seem excessive and also that “of concern is the mean anticholinergic effect of 2.4. Some supportive reference for viewing these scores as such would be helpful.  

**We hope we have clarified this. We provide information on links between anticholinergic effect on cognition with mental wellbeing later in the referenced paragraph. We state that we use the scoring system described by (Bishara et al., 2017) in the sections: ‘Health status and perceptions of physical wellbeing’ and ‘Data collection’.**

 Pg 12 - It was proposed that a larger sample with patients aged over 18 may have led to different findings. I wasn’t sure about what point was being made by this sentence. Some clarification would be helpful. 

 + 

 Pg 12 - It was reported that some patients may have felt younger and rated a number of outcomes in line with this. If this is the case, how valid are the measures used? 

**In response to the two points above – we agree that this is confusingly worded. We have sought to simplify this by deleting the following, and simplifying our main point in this paragraph:**

**“As our outcomes were largely subjectively measured it is possible some respondents felt younger or older than others the same age and rated their mental wellbeing, physical health and recovery-related quality of life in line with this. A larger sample with patients aged 18 and older might have led to different findings.”**

****

 Implications 

 On pg12, it is stated that our data suggest that older patients could benefit from interventions to improve cognition or ameliorate cognitive decline. This needs to be clearly detailed from the data as, at present, this doesn’t seem to be the case – the data only states there is cognitive decline in these participants and it is in the discussion where ways of dealing with this are suggested. 

**We agree implications should be couched in sufficient evidence. We have reworded this section to reflect our findings and signpost the need for more research:**

**“To summarise the practical implications of our study, 65% of our sample had possible cognitive impairment according to a validated measure. This suggests that older patients might benefit from interventions to improve cognition or ameliorate cognitive decline, though more evidence is needed to speak to the efficacy of different interventions. Studies suggest improvement might be best achieved through cognition-based interventions, physical exercise and antioxidants ( Xu et al., 2021).”**

 Limitations 

 These need to be clearly noted. There are issues reading sample size and it is questionable whether the study was sufficiently powered for some of the correlational analysis. The representativeness of the sample and how generalisability of the findings should also be considered 

**This is an important point. We have acknowledged this by including the following in the ‘Limitations’ section:**

**“Finally, we did not compare our sample to a representative or whole population of all older patients in England and Wales or a representative or whole population sample of all patients; nor did we obtain a random sample of participants. This places limitations on the extent to which we can conclude our findings are generalisable to patients outside our sample.”**

 Conclusions 

 It is suggested on pg13 services co-develop with patients’ meaningful activities. I agree with this statement but if it is to be a concluding point, it should be explicitly mentioned in the discussion (or elsewhere in the paper). 

**We are glad that the Reviewer agrees that this an important point. As we do not think we can introduce the topic of co-production in detail in this manuscript, we have made a small reference to this in the introduction, as we would like to keep this in:**

**“Investigating and documenting the disparities between younger and older mental healthcare patients can better equip us to shape service provision, co-develop responsive and appropriate interventions with patients, and address structural disparities in health and wellbeing outcomes ( Hui et al., 2021).”**
